# Methacrylation of Fibrillar Sea Urchin Collagen: Production of Sustainable, Stable, and Functional Hydrogels

**DOI:** 10.1021/acsomega.6c01928

**Published:** 2026-07-07

**Authors:** Margherita Roncoroni, Giordana Martinelli, Tamara Chwojnik, Chiara Scapuzzi, Luca Melotti, Anna Carolo, Daniela Maggioni, Stefano Farris, Marco Patruno, Stefania Marzorati, Michela Sugni

**Affiliations:** † Department of Environmental Science and Policy, 9304 University of Milan, Via Celoria, 2, 20133 Milan, Italy; ‡ Department of Comparative Biomedicine and Food Science, University of Padua, Via dell’Università, 16, 35020 Legnaro, PD, Italy; § Department of Chemistry, University of Milan, Via Golgi 19, 20133 Milan, Italy; ∥ Department of Food, Environmental and Nutritional Sciences, University of Milan, Via Celoria, 2, 20133 Milan, Italy

## Abstract

Marine-derived biomaterials represent a sustainable alternative to mammalian sources for biomedical applications, aligning with circular economy principles. In this study, native fibrillar collagen extracted from food waste of the sea urchin*Paracentrotus lividus* was successfully methacrylated for the first time to produce structurally stable, photo-cross-linkable hydrogels. A dedicated methacrylation protocol was developed to overcome the intrinsic limitations imposed by the supramolecular organization of intact collagen fibrils, enabling effective functionalization while preserving their native architecture. Methacrylated collagen hydrogels were obtained *via* UV-induced photo-cross-linking and further functionalized with sea urchin–derived polyhydroxynaphthoquinones to generate antioxidant composite hydrogels. The resulting biomaterials were comprehensively characterized in terms of ultrastructure, macroporosity, swelling behavior, mechanical properties, water uptake, degradation kinetics under physiological and enzymatic conditions, antioxidant activity, and *in vitro* cytocompatibility using human dermal fibroblasts. Compared with non-methacrylated collagen scaffolds, methacrylated hydrogels exhibited enhanced structural stability, reduced swelling-induced deformation, and significantly increased resistance to degradation. Incorporation of sea urchin-derived antioxidants further improved hydrogel stability and conferred marked antioxidant activity, which was largely preserved after incorporation. *In vitro* assays demonstrated that the hydrogels supported cell viability and metabolic activity. Overall, these findings demonstrate that methacrylated sea urchin collagen hydrogels, with or without antioxidant loading, constitute a promising class of sustainable biomaterials for tissue engineering applications.

## Graphical abstract

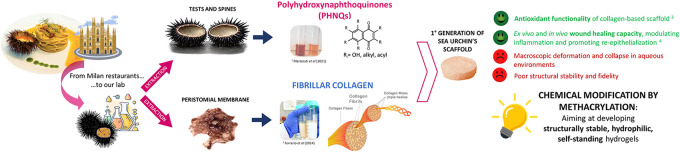

## 1 Introduction

Over the past decade, marine (blue) biotechnology has focused on the sustainable exploitation of marine biodiversity to obtain high-value molecules, bioactive compounds, and biomaterials for healthcare,^1^ regenerative medicine,^2^ and related biotechnological sectors.^3–5^ For example, marine-derived biomaterials made of sodium alginate and chitosan have gained increasing attention due to their biocompatibility, biodegradability, and functional versatility.^6,7^ Alginate, extracted from brown seaweeds, is widely used in hydrogel systems, while chitosan, derived from crustacean exoskeleton, is valued for its antimicrobial and wound-healing properties. The possibility to recovery large amounts of these polymers from byproducts highlights the potential of marine resources as sustainable platforms for advanced biomedical applications, aligning with circular economy principles and minimizing waste and environmental impact.^8,9^ In this context, sea urchins turned out to be a promising source of biomolecules for “eco-friendly” biomedical research. These animals produce potent antioxidant compounds (*i.e.*, polyhydroxynaphthoquinones-PHNQs);^10,11^ and possess unique dynamic connective tissues (*i.e.*, mutable connective tissue-MCTs), which can be a valuable source of native collagen fibrils.^12–14^ Indeed, the intrinsic nature of MCT allows to “easily” obtain high yields of collagen fibrils fully preserving their structural integrity, including the surface decoration of glycosaminoglycans (GAGs).^15^ In an applicative perspective, this likely enhances mechanical strength and bioactivity, making sea urchin’s collagen a promising candidate for tissue engineering.^15–17^ The extraction of added-value compounds, namely antioxidants and native fibrillar collagen, from the food waste of edible Mediterranean sea urchins (*i.e.*, *Paracentrotus lividus* and *Sphaerechinus granularis*) has already been shown to be an effective way of converting marine byproducts into valuable biomolecules for use in biomedical applications.^15,18–24^ This approach valorizes waste while promoting environmentally friendly and biomimetic biomaterials for tissue regeneration.

Tissue engineering in fact increasingly relies on biomaterials capable of mimicking the native extracellular matrix and providing appropriate structural and biochemical cues to guide tissue repair and functional restoration. In particular, scaffolds derived from marine resources are gaining interest due to their biocompatibility, bioactivity, and structural similarity to natural tissues. Materials such as collagen, alginate, and chitosan can be engineered into hydrogels, membranes, and three-dimensional porous constructs that support cell adhesion, proliferation, and differentiation, while enabling controlled degradation and integration with host tissues. These features make marine-derived biomaterials especially suitable for applications including skin wound healing and soft tissue repair, further reinforcing their role as sustainable and high-performance candidates for next-generation regenerative therapies.^25–27^

Collagen is particularly suitable for the development of biomimetic scaffolds that could integrate well with host tissues,^28–30^ being the most abundant structural protein of the extracellular matrix and having an essential role in tissue architecture, cell adhesion, migration, differentiation, and regeneration.^31,32^ A previous work showed that collagen from *P. lividus* can be used to fabricate 2D membranes and 3D porous scaffolds with *in vitro* biocompatibility and ability to support cell infiltration,^18^ as well as *ex vivo* and *in vivo* wound healing capacity by modulating inflammation and promoting re-epithelialization.^21,23^ Moreover, sea urchin PHNQs have also been extensively studied for their antioxidant and antibacterial properties.^20,33–37^ Recently, PHNQs have been incorporated into collagen-based biomaterials to enhance functionality, improving epidermal cells migration in moist wound environments in *ex vivo* models while promoting biomaterial structural integrity.^24,38^

Nevertheless, sea urchin-derived collagen scaffolds tend to undergo macroscopic deformation in aqueous environments, including collapse of their 3D structure,^19,38^ which might limit handling and performance. In fact, for sustainable materials to be impactful, improved environmental sourcing must be coupled with quantifiable gains in performance and durability. In this context, enhancing the structural stability and degradation resistance of waste-derived marine collagen is critical to justify its substitution for conventional collagen systems in biomedical applications.

To address this issue and as a step forward, the present study focused on developing structurally stable sea urchin-derived biomaterials in the form of hydrogels: three-dimensional, hydrophilic, self-standing matrices formed by cross-linked polymers capable of retaining large amounts of water.^39,40^ In collagen-based hydrogels, cross-linking is crucial to improve mechanical stability and structural fidelity.^41,42^ Traditional cross-linking methods—such as UV irradiation or chemical agents like glutaraldehyde and carbodiimides—are widely used for hydrolyzed collagen^43^ but may leave behind cytotoxic byproducts, alter collagen structure, or compromise biocompatibility.^44^

In this context, methacrylation was chosen as a more controlled and versatile alternative. This method introduces methacrylate groups into collagen. This allows photo-cross-linking under mild, aqueous conditions using a photoinitiator under UV or visible light.^45^ The result is a covalently cross-linked network with improved mechanical stability and a controlled degradation rate,^46,47^ which is ideal for tissue engineering.^46,48^ Methacrylation also offers precise control over cross-linking degree, is compatible with 3D printing, and support the preservation of the native structure of the biomaterial.^45,49^

In literature, methacrylated collagen is most commonly obtained from mammalian gelatin, a partially hydrolyzed collagen, where the fibrillar supramolecular organization is irreversibly lost.^50^ While widely available, this mammalian source raises ethical and environmental concerns^51,52^ as well as potential risks relating to allergic reactions and the transmission of serious diseases, such as bovine spongiform encephalopathy.^53^ In contrast, marine collagens, such as those extracted from sea urchin waste, offer a potentially safer, sustainable, and ethically viable alternative.

Based on these considerations, this study aimed to develop methacrylated collagen (CollMA) hydrogels from fibrillar sea urchin collagen. Since this collagen has not been hydrolyzed during the extraction protocol, and so it kept its supramolecular quaternary structure (the native fibril composed of aligned tropocollagen triple-helixes), a dedicated methacrylation protocol needed to be developed for the purpose for the first time, addressing the challenges of ensuring methacrylation efficiency posed by the hindrance in mass transfer processes due to the presence of macroscopic fibrillar structures in suspension (rather than isolated peptides in solution). Ultimately, the development of methacrylated native collagen hydrogels represents a step toward more biomimetic and sustainable biomaterials with controllable mechanical and biological properties. Given their previously demonstrated ability to enhance bioactivity and structural stability,^38^ PHNQs were also incorporated into the new hydrogels to promote any further functional improvement.

The resulting hydrogels—with and without incorporation of sea urchin antioxidants (PHNQs/CollMA and CollMA)—were characterized in terms of ultrastructure *via* SEM, porosity, swelling behavior, mechanical properties, degradation kinetics, and *in vitro* cytotoxicity, and compared with non methacrylated sea urchin collagen scaffolds (Coll).

## 2 Materials and Methods

### 2.1 Sea Urchins Waste Recovery

*P. lividus* waste, after gonads removal, was collected from local restaurants near the University of Milan. The waste was immediately frozen and stored at −20 °C.

### 2.2 Extractions

#### 2.2.1 Collagen

Collagen was extracted as reported by Ferrario and colleagues.^16,18^ Peristomial membranes were isolated, rinsed with artificial seawater, and incubated overnight at 23 °C in hypotonic buffer (10 mM Tris–HCl, pH 8.0; 0.1% EDTA). After PBS washes, tissues were decellularized overnight at 23 °C in 10 mM Tris–HCl, 0.1% SDS (pH 8.0), then transferred to a disaggregating solution (0.5 M NaCl, 0.1 M Tris–HCl, pH 8.0; 0.1 M β-mercaptoethanol; 0.05 M EDTA) and stirred for 5 days at 23 °C. The collagen suspension was filtered (0.2 μm steel mesh), dialyzed for 4 h against 0.5 M EDTA and overnight against distilled water, then stored at −80 °C. Collagen suspension concentration (mg/mL) was obtained by lyophilizing and weighing a 1 mL aliquot.

#### 2.2.2 PHNQs

PHNQs were extracted according to Marzorati and co-workers.^20^ Briefly, lyophilized test and spine powders were treated with 6 M formic acid to dissolve the carbonate matrix and stirred for 2 h at room temperature. After centrifugation (6000*g*, 5 min, Eppendorf, Centrifuge 5804 R), the supernatant was vacuum-filtered and subjected to triple liquid–liquid extraction with ethyl acetate. The organic phase was collected and further purified by repeated washes with Milli-Q water to remove inorganic salts. The combined organic phases were washed with Milli-Q water until neutral pH was reached, anhydrous sodium sulfate was added, the solution was filtered, and finally the extract was dried using a rotary evaporation (37 °C, Buchi, Italia srl).

### 2.3 Hydrogels Production

#### 2.3.1 Collagen Methacrylation

Fibrillar collagen methacrylation was achieved by modifying *ad hoc* a previously published protocol developed for hydrolyzed collagen.^54^ In details, a precise volume of sea urchin fibrillar collagen suspension was centrifugated and resuspended in water or buffer and reacted with methacrylic anhydride (MA, 94%, stabilized with approximately 0.2% 2,4-dimethyl-6-*tert*-butylphenol, Thermo Fisher, United States). The reaction was carried out in the dark under magnetic stirring at room temperature. The resulting suspension was then dialyzed (dialysis tube cellulose membrane, Sigma-Aldrich, Germany) against distilled water at 4 °C to facilitate the removal of unreacted methacrylic anhydride, buffer and other reaction byproducts. The final methacrylated collagen suspension was stored at −80 °C until use.

To develop the most effective protocol for methacrylation while maintaining the stability of the resulting suspension, different parameter ranges were tested and optimized. Details on the experimental trials are reported in Tables 1 and S1 in the Supporting Information.

**Table 1:** Different Parameter Ranges Tested to Develop the Most Effective Protocol for Collagen Methacrylation

parameter	tested parameter ranges
Collagen concentration (mg/mL)	4–5
Na_2_HPO_4_ concentration in the solvent mixture (M)	0–0.2
Volume of Methacrylic anhydride MA (mL) per mL of collagen suspension	0.129–0.645
Reaction time (h)	4–6
Dialysis time (days)	5–10 days

Once the reaction conditions were optimized, commercial standards of free amino acids bearing available amine groups not involved in peptide bond formation (namely lysine, arginine, and histidine, from Merck) were subjected to the same protocol to assess their relative reactivity and verify the effectiveness of the methacrylation reaction. Experimental details and full analyses are provided in the Supporting Information (Figures S2 and S3).

#### 2.3.2 Methacrylated Collagen Hydrogels (CollMA)

Methacrylated collagen hydrogels (CollMA) were produced starting from the previously obtained methacrylated collagen suspension, by a cross-linking reaction step using 2-hydroxy-4′-(2- hydroxyethoxy)-2-methylpropiophenone as a photoinitiator (98%, Irgacure 2959, Merck (Germany)). In details, 33 μL of a methanolic solution of Irgacure (135 mM) were added to 1 mL of the methacrylated collagen suspension (10 mg/mL) to achieve a final Irgacure content of 10% by weight of collagen. One mL of the resulting mixture was poured into a cylindrical mold (1 cm diameter) and exposed to UV irradiation at 365 nm (UV Exposure System, UV lamp Model No. KGW9N_UVA PL Lamp 9W × 4 EA) for different durations (5, 10, or 30 min) to identify the exposure time required for complete hydrogel formation. The samples were frozen at −80 °C and freeze-dried (Edwards Pirani 1001) overnight (CollMA). After lyophilization, they were extensively washed with water to remove Irgacure (and any other unreacted compound) and subsequently freeze-dried again.

#### 2.3.3 Methacrylated Collagen + PHNQs Hydrogels (PHNQs/CollMA)

Aiming at adding antioxidant functionality to methacrylated collagen-based hydrogels (CollMA), PHNQs were incorporated to produce composite hydrogels (PHNQs/CollMA). Specifically, 250 μL of a PHNQs aqueous solution were dropped onto previously lyophilized CollMA scaffold, ensuring complete absorption. PHNQ solution was prepared to achieve a PHNQ loading of 10% w_PHNQ_/w_collagen_. The resulting scaffolds were subsequently freeze-dried again.

#### 2.3.4 Control Collagen Scaffolds (Coll)

Control collagen scaffolds (Coll) were prepared by resuspending in a 6% (v/v) EtOH/water solution the collagen suspension (*i.e.*, not methacrylated) to obtain a final concentration of 10 mg/mL.^16^ A 1 mL aliquot of this collagen suspension was then poured into silicone rubber molds (diameter: 1 cm), frozen at −80 °C and lyophilized overnight (Edwards Pirani 1001).

#### 2.3.5 Collagen Hydrolysis

Coll and CollMA were hydrolyzed in order to obtain solutions that could be analyzed by ^1^H NMR and subjected to the ninhydrin assay. Briefly, a method by Benedetto et al.^15^ was modified as follows: 1 mL of a 15 mg/mL stock Coll or CollMA suspension (in distilled water) was centrifuged (10,000*g*, 15 min, 4 °C), the supernatant was discharged and resuspended in 1 mL of pepsin (1 mg/mL) in acetic acid 0.5 M. Samples were left 5 days (4 °C) on an rotary shaker, until clear solutions were obtained.

### 2.4 Physico-Chemical Characterization of Methacrylated Collagen

#### 2.4.1 Fourier Transform Infrared Spectroscopy

An Attenuated Total Reflection-Fourier Transform Infrared (ATR-FTIR) spectrometer (Spectrum 100, PerkinElmer) was used to record spectra of methacrylated collagen and control collagen. Spectra of the air-dried samples were recorded in the 4000–1100 cm^–1^ range with 8 scans.

#### 2.4.2 Ninhydrin Assay

Hydrolyzed solution of Coll was serially diluted in water in the range 1–10 mg/mL in triplicate. Hydrolyzed CollMA solution was used at 7.5 mg/mL without dilution (in triplicate). Modifying a literature method,^55^ a 12 mM (2.2 mg/mL) solution of ninhydrin (from Merck) in ethanol was added to each sample in a 1:8 v/v ratio of ninhydrin to collagen solution. Samples were sealed and incubated in an oven at 70 °C for 30 min. Absorbance was measured at 570 nm using a spectrophotometer (Hanna). The mean absorbance for each Coll standard solution was plotted to obtain a standard curve. For CollMA sample, the fraction of amines available was determined by calculating the ratio between the apparent sample concentration (read from the calibration line) and the nominal concentration. Finally, the DoF (degree of functionalization) was determined as

$$DoF\;\left(\%\right)=100\times \left(1-\frac{apparent\;sample\;conc.}{no\min al\;sample\;conc.}\right)$$

#### 2.4.3 Nuclear Magnetic Resonance (NMR) Spectroscopy

The NMR spectra were acquired in aqueous solution (Bruker NEO400 spectrometer) equipped with a Bruker BBI Z-gradient reverse probe head with a maximum gradient strength of 53.5 G/cm (π/2 pulse: ^1^H 8.0 μs) operating at 400.43 MHz to investigate the reactivity of amino acids (See Supporting Information) and the metacrylation degree of CollMA after an hydrolysis treatment, as well as in the solid state on the native Coll, CollMA and on UV-cross-linked CollMA to confirm the efficacy of methacrylation reaction (Bruker DRX500 spectrometer equipped with a Bruker 5 mm BBI Z-gradient probe head with a maximum gradient strength of 53.5 G/cm (π/2 pulse: ^1^H 8.5 μs) operating at 400.13 MHz).

### 2.5 Hydrogels Characterization

The obtained methacrylated collagen hydrogels (CollMA) and PHNQs-loaded methacrylated collagen hydrogels (PHNQs/CollMA) were characterized and compared with control collagen scaffolds (Coll).

#### 2.5.1 Ultrastructure (SEM)

The ultrastructure of the samples was investigated by scanning electron microscopy (SEM). Lyophilized hydrogels and scaffolds were mounted on aluminum stubs, gold coated (Leica ACE600 sputter coater, Leica Microsystems, Germany) and observed and photographed under a FE-SEM Sigma (ZEISS, Germany) scanning electron microscope.

#### 2.5.2 Macroporosity

Macroporosity was calculated using the water squeezing method.^56^ Briefly, scaffolds were immersed in PBS for 1 h, weighed (*P*_1_), squeezed, and reweighed (*P*_2_). The macroporosity volume was calculated using the following formula

$$\mathrm{macroporosity}\;\left(\%\right)=\frac{\left(P_{1}-P_{2}\right)}{P_{1}}\times 100$$

The experiment was repeated 5 times.

#### 2.5.3 Swelling and Water Uptake Properties

The lyophilized samples were photographed with a digital camera and then 5 mL of PBS 1× were added at 37 °C for 3 h. The samples were then photographed again and the percentage changes in thickness and area were measured by comparing the pre- and postwetting photographs using ImageJ software. In addition, water uptake was determined by evaluating the percentage change in weight before and after 3 h of swelling in distilled water at 37 °C. Prior to weighing, excess surface water was carefully removed using absorbent paper. The experiment was repeated 5 times.

#### 2.5.4 Degradation Kinetics

The degradation test was performed in both PBS 1× and aqueous collagenase solution. Lyophilized hydrogels were weighed, and 5 mL of PBS or 0.01 mg/mL collagenase solution was added (50 mM Tris, 100 mM NaCl, 10 mM CaCl_2_, collagenase enzyme type I from *Clostridium histolyticum*, Sigma-Aldrich, Germany). To mimic wound environment the hydrogels were kept at 37 °C. At defined time points (PBS: 1, 7, 10 days; collagenase: 6–168 h), samples were washed, frozen (−80 °C), lyophilized, and reweighed to determine the percentage of residual mass, calculated using the following formula

$$\mathrm{mass}\;\mathrm{remaining}\;\left(\%\right)=\frac{\left(\mathrm{initial}\;\mathrm{weight}-\mathrm{final}\;\mathrm{weight}\right)}{\mathrm{initial}\;\mathrm{weight}}\times 100$$

In both conditions (PBS and collagenase enzyme), 4 replicates were used for each time point.

#### 2.5.5 Antioxidant Activity

The antioxidant activity of PHNQs/CollMA hydrogels was evaluated using an ABTS assay previously optimized in our laboratory.^20^ The ABTS radical cation (ABTS^•+^) was generated by reacting 7 mM ABTS with 2.45 mM ammonium persulfate and allowing the mixture to stand overnight in the dark at room temperature. The resulting ABTS^•+^ solution was then diluted in ethanol (1:75 v/v) to obtain an absorbance of ∼0.7 at 734 nm.

Hydrogels were immersed in PBS for 1 or 10 days before testing. Different and increasing weights of lyophilized samples (range 0.2–2 mg) were then incubated with the ABTS^•+^ solution, and the antioxidant activity results were compared with those of CollMA at *t* = 0 (without PHNQs) and of PHNQs solubilized in PBS, to determine whether the antioxidant activity of hydrogel-loaded PHNQs matched that of free pigments.

Samples were incubated in the dark for 1 h, and absorbance at 734 nm was measured using a spectrophotometer (Hanna). A blank solution without sample was used as reference. The percentage of remaining ABTS^•+^ was calculated as

$${\%\mathrm{ABTS}}^{•+}\;\mathrm{remaining}=\frac{A_{734\mathrm{nm},1h,\mathrm{sample}}}{A_{734\mathrm{nm},1h,\mathrm{blank}}}\times 100$$

#### 2.5.6 Mechanical Tests

Scaffolds (*n* = 3) were evaluated in a typical compression test after being swelled with 500 μL distilled water for 5 min. Tests were only conducted on CollMA and PHNQs/CollMA specimens because the Coll collapsed immediately upon contact with water. According to our previous work,^38^ two consecutive compression cycles were performed using a dynamometer (mod. Z005, Zwick Roell, Ulm, Germany) fitted with a 100 N load cell and connected to two plates (base plate: 150 mm diameter; compression plate: 30 mm diameter) placed at a distance of 22 mm apart. Each compression cycle accounted for a maximum deformation of the sample of 2 mm, at a speed of 2 mm s^–1^. A force–time profile was recorded, from which two different parameters were then acquired: (i) resistance to compressive stress (*R*_max_), expressed as maximum compressive force in N and (ii) elastic recovery (*E*_rec_), expressed as the difference in compression energy (J/m^2^) between first and second compressive cycle. Data were elaborated by TestXpert V10.11 Master software.

### 2.6 Cytocompatibility Test

#### 2.6.1 Normal Human Dermal Fibroblasts Cell Culture and Biomaterial Seeding

Normal human dermal fibroblasts (NHDF) were cultured in Dulbecco’s Modified Essential Medium (DMEM; Sigma Merck, Darmstadt, Germany) low glucose (1 g/L) with l-glutamine (2 mM) supplemented with 15% (w/v) fetal bovine serum (FBS) (Sigma Merck, Darmstadt, Germany), and antibiotic antimycotic solution composed of penicillin (100 U/mL), streptomycin (10 mg/mL), and amphotericin B (25 ug/mL) (Sigma Merck, Darmstadt, Germany). Cells were cultured at 37 °C in a humified 5% (v/v) CO_2_ incubator. Cells were refreshed with new cell culture medium every 2–3 days and passaged at subconfluence. All experiments were performed with cells at passage 3–4.

Prior to cell seeding, CollMA hydrogels were exposed to UV for at least 30 min to ensure proper sterility. Afterward, they were rehydrated and washed twice in sterile UP water (Sigma Merck, Darmstadt, Germany), then acclimated in complete cell culture medium for 2 h in a humified 5% (v/v) CO_2_ incubator at 37 °C. Afterward, cells were seeded at a density of 3 × 10^5^ cells/cm^2^; cell solution was added dropwise to the biomaterial and left to incubate for 1 h at 37 °C. Then, complete cell culture medium was added to the cell culture well plate to each specimen. Cell culture medium was refreshed every 3 days. CollMA hydrogels were tested together with a low-methacrylate collagen hydrogel (CollMA2), synthesized with a substantially reduced methacrylic anhydride content (5.7 μL MA/mL of collagen suspension). CollMA2 hydrogels were subjected to the same procedures for sterilization and cell seeding as described for CollMA.

#### 2.6.2 NHDF Cell Viability Assay (Cytocompatibility Test) and Cell Morphology

The alamarBlue HS Cell Viability (ThermoFisher Scientific, Waltham, MA, US) assay was used to assess cell viability. At each time point (1, 3, 5, and 7 days), samples were washed twice with PBS and incubated with 500 μL of alamarBlue solution (10% v/v, diluted in cell culture medium). Samples were kept in a humified chamber with 5% CO_2_ at 37 °C for 4 h protected from light. Eventually, aliquots of 100 μL of supernatant were distributed in a 96-well cell culture plate in quadruplicates. Plates were read using a microplate reader (Varioskan LUX Multimode Microplate Reader, ThermoFisher Scientific, Waltham, MA, US) at λ = 544/590 nm (ex/em); hydrogels without cells and kept in culture in standard conditions were used as blanks whereas NDHF seeded onto cell plastic support were used as positive controls. The experiments were repeated at least three times.

To investigate cell morphology, samples were washed with PBS and fixed using 4% (v/v) paraformaldehyde for 15 min at room temperature. Afterward, samples were permeabilized with Triton-X (0.5% v/v) in PBS for 20 min at room temperature. For staining the f-actin filaments, phalloidin toxin conjugated with TRITC was used (Thermo Fisher Scientific, Waltham, WA, USA) at a concentration of 50 μg/mL; samples were incubated for 30 min in the dark. Then, nuclei were stained using Hoechst 33342 (1 μg/mL; Sigma Merck, Damstadt, Germany). Samples were observed using the fluorescent microscope (Leica Thunder Imager, Leica, Wetzlar, Germany).

### 2.7 Statistical Analyses

Macroporosity and hydration data (*n* = 5) were analyzed using one-way ANOVA, as they followed a normal distribution (Shapiro–Wilk and Kolmogorov–Smirnov, *p* > 0.05). Water uptake data (*n* = 5) were non-normal (*p* < 0.05), so the Kruskal–Wallis test with Dunn’s posthoc was applied. MANOVA was used to assess the significance of differences in degradation kinetics (*n* = 4) under physiological and enzymatic conditions as it allows simultaneous consideration of multiple factors (treatment and time) and their interaction effects, which could not be adequately addressed by nonparametric alternatives. All statistical analyses were performed in SPSS.

Statistical analyses for data (mean ± SD) regarding cell viability were performed using XLSTAT software package (Data Analysis and Statistical Solution for Microsoft Excel, Addinsoft, Paris, France). Comparison among days were performed using the Kruskal–Wallis nonparametric test; p values less or equal to 0.05 were considered statistically significant.

## 3 Results

### 3.1 Optimization of Collagen Methacrylation Reaction

Figure 1 shows a schematic representation of the scaffold production, from the collagen fibril suspension methacrylation, to the final cross-linked biomaterial.

**Figure 1:**
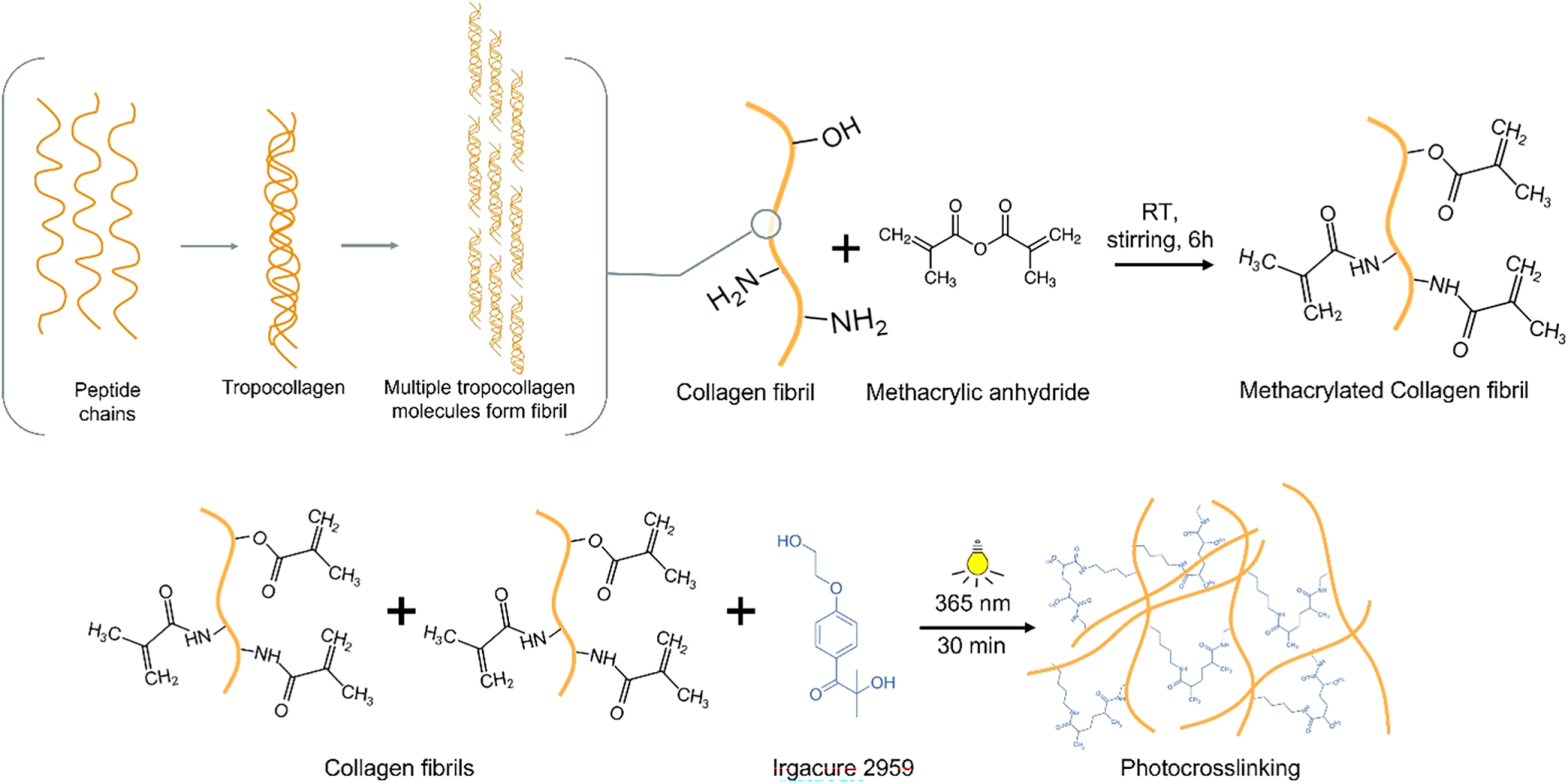
Schematic representation of the methacrylation and photo-cross-linking process of sea urchin-derived collagen. The free amine and OH groups on the collagen fibrils react with the methacrylic anhydride at room temperature when stirred for 6 h, which introduces the methacrylate moieties. The modified collagen is then mixed with the photoinitiator Irgacure 2959, after which it is exposed to UV light (365 nm) for 30 min to induce photo-cross-linking and the formation of a hydrogel network.

Methacrylation optimization involved several trials to establish the optimal combination of the parameters: fibrillar collagen concentration, concentration of Na_2_HPO_4_, collagen/methacrylic anhydride ratio, reaction time and the most appropriate dialysis period for purifying the methacrylated collagen from unreacted precursors and reaction byproducts. Non optimal conditions leading to negative results (*e.g.*, collagen aggregation or uncomplete hydrogel formation) were excluded and considered ineffective. Overall, all the protocol optimization tests were necessary to obtain a product that was sufficiently stable from a structural point of view while suitable for the potential biomedical applications.

Cross-linking also needed optimization trials to define the optimal UV exposure time to obtain a fully formed hydrogel. Optimized conditions are reported in Table 2, ensuring the formation of a well-formed hydrogel.

**Table 2:** Optimized Condition for the Successful Preparation of Structured Hydrogels

parameter	tested parameter ranges	optimized parameters
Collagen concentration (mg/mL)	4–5	5
Na_2_HPO_4_ concentration in the solvent mixture (M)	0–0.2	0.2
Volume of Methacrylic anhydride MA (mL) per mL of collagen suspension	0.129–0.645	0.129
Reaction time (h)	4–6	6
Dialysis time (days)	5–10 days	10

Then, the optimized methacrylation protocol was performed on individual amino acids and the corresponding ^1^H NMR analyses (see Figures S2 and S3 in Supporting Information) confirmed that, among the amino acids bearing free amine groups, only lysine residues were effectively methacrylated.

ATR-FTIR spectra (Figure 2) were acquired to evaluate the successful incorporation of methacrylate functionalities by comparing methacrylated collagen suspension (CollMA) with unreacted sea urchin collagen (Coll), used as a control.

**Figure 2:**
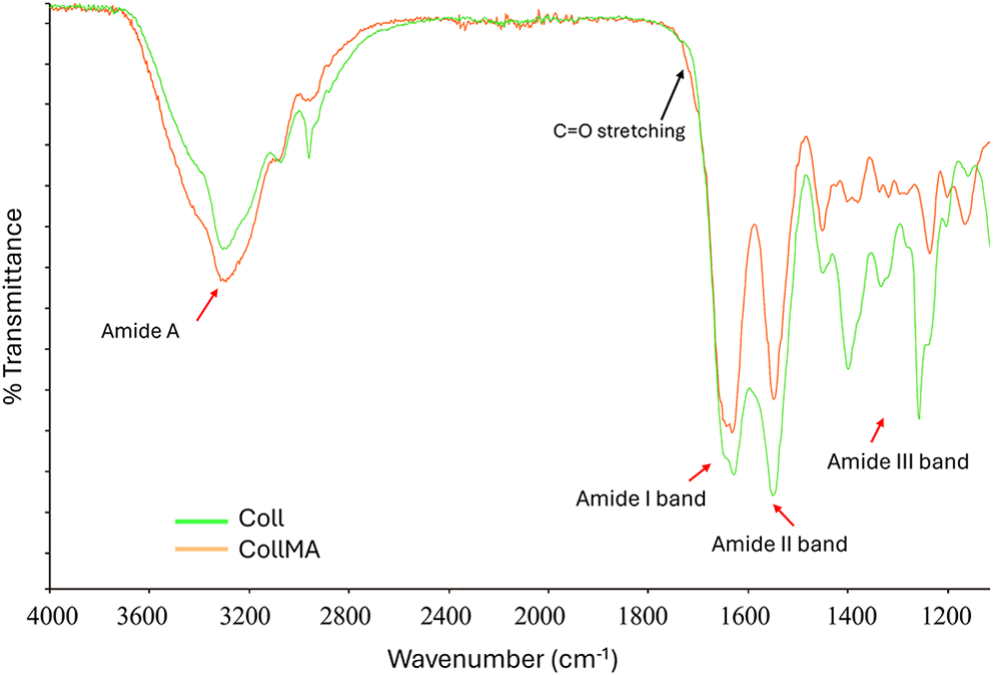
ATR-FTIR spectra of standard collagen (Coll, green) and methacrylated collagen (CollMA, orange).

As expected and based on previous literature,^57^ the pristine unreacted collagen spectrum (green line in Figure 2) is characterized by the presence of several bands attributable to collagen functional groups. Strong bands are present between 1700 and 1200 cm^–1^ attributable to (i) amide I at ∼1650 cm^–1^ (results from the stretching vibration of the peptide carbonyl group (−C=O));^58–60^ (ii) amide II at ∼1560 cm^–1^ (attributed to NH bending vibration coupled with CN stretching);^58,61^ (iii) amide III vibration modes centered at ∼1245 cm^–1^ (which corresponds to the stretching vibrations of C–H).^62^ The peak of the amide III band indicates collagen with a complete triple helix structure.^63^

In CollMA (orange line in Figure 2) these bands decreased or were modified, indicating reduced chain mobility due to covalent attachment of methacrylate groups;^38^ a weak shoulder at ∼1700 cm^–1^, attributable to ester C=O stretching, further supported a reaction which may have also involved the hydroxyl groups of specific amino acid residues. Nevertheless, the ATR-FTIR spectra were insufficient to well-explain methacrylation efficacy in the collagen samples.

Therefore, NMR spectroscopy was taken into account and, due to the insolubility of sea urchin native collagen, the analyses were carried out in solid state exploiting for ^13^C NMR spectra the CPMAS experiments. Figure 3 shows the comparison between the ^13^C CPMAS NMR spectra of the pristine sea urchin collagen (Coll, green trace), the methacrylated collagen suspension (CollMA suspension, orange trace), and the cross-linked collagen after UV irradiation (CollMA, red trace). The ^13^C CPMAS NMR spectrum of the pristine collagen shows the ^13^C signals of the major amino acids (Hip, Pro, Glu, Ala, and Gly) in the region of the methylene and methyl signals, in agreement with the attributions reported in the literature.^64^ Also, it is recognizable the quaternary carbon signal relative to the guanidinium group of arginine (Arg ε). After the collagen is reacted with methacrylic anhydride, new signals are visible in the aliphatic region, attributed to the different acrylic methyl groups present on different amino acids and, hence, lying in different magnetic environments, while the methylene groups of the methacrylate moiety were barely recognizable at ca. 130 ppm due to their poor intensity. After the photochemical cross-link of the methacrylate groups was performed, the signals of amino acids residues enlarged and overlapped. The broadening of the bands is correlated to the shortening of the relaxation times, which in turn indicate a greater rigidity of the system caused by the cross-linking of the methacrylated coils. Also, after the cross-linking a shift of part of the carbonyl groups is visible in the ^13^C spectrum.

**Figure 3:**
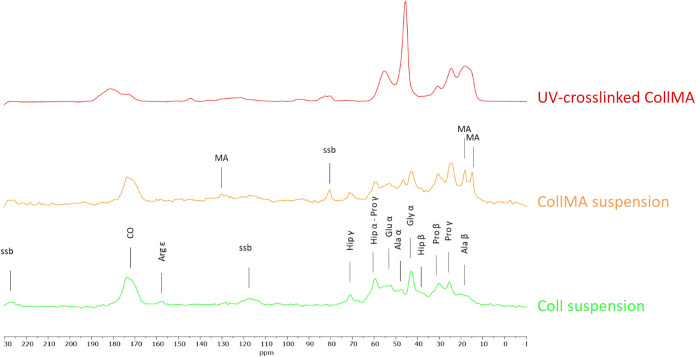
Solid state ^13^C CPMAS NMR spectra of pristine collagen suspension (Coll, green), methacrylated collagen suspension (CollMA, orange) and cross-linked collagen after UV photo-cross-linking (red trace).

Given the qualitative confirmation of successful methacrylation, the degree of functionalization (DoF) was assessed by means of ^1^H NMR and ninhydrin assay, providing quantitative information about the availability of reactive groups and, consequently, the cross-linking potential of the system. These measurements required Coll and CollMA to be hydrolyzed in order to obtain analyzable solutions.

The ninhydrin assay, specific for the detection of amine groups, showed a DoF of 7%_w/w_, confirming the limited functionalization of the collagen chains. This result is consistent with the low lysine content of sea urchin-derived collagen, as lysine is the only amino acid bearing an available NH_2_ group that can be methacrylated. Since lysine accounts for a relatively low fraction (0.86%_w/w_) of the amino acids in sea urchin collagen,^38^ the limited availability of reactive sites leaded to a moderately low degree of methacrylation and explains the long UV exposure time required for cross-linking.

On the other hand, it is known that gelatin, the hydrolyzed form of collagen, can react with both NH_2_ residues, forming methacrylamide groups, and OH residues, giving rise to methacrylate groups.^65^ Hence, in order to quantify the degree of methacrylation that takes into account also the OH residues, ^1^H NMR spectroscopy was employed, acquiring 1D spectra of native collagen (Coll) and methacrylated one (CollMA) after hydrolysis through the treatment with pepsin. Figure S4 shows the comparison between the two ^1^H spectra acquired in a H_2_O/D_2_O 9:1 solution, where the water signal was partially suppressed. The spectra also contain signals from acetic acid (*), deriving from the hydrolysis process. The CollMA spectrum shows, in the olefinic region, a first set of signals at 6.01 and 5.62 ppm, assigned to methacrylate groups,^65^ likely deriving from the reaction with hydroxyproline, threonine, serine, and tyrosine residues, which are present in Coll at a total amount of 19.4% _w/w_ (see Table S2 and the calculations reported in the Supporting Information). A second minor set of olefinic signals at 5.57 and 5.33 ppm was assigned to methacrylamide groups,^65^ originating exclusively from the reaction of lysine amine residues (0.86%_w/w_). By taking as an internal standard the integrated intensity of the methyl signals of leucine and valine lying in the 0.73–0.91 ppm range, and considering their weight percentage content (2.96% and 2.50%, respectively, corresponding to 0.02256 and 0.02134 mol per 100 g of Coll),^38^ it was possible to estimate that more than 95% of lysine residues were functionalized, whereas only 10.5% of the OH groups present in the aforementioned four amino acids were methacrylated, despite their much higher abundance that is due to the much less OH nucleofilicity compared to the one of amine groups. Overall, the total amount of methacrylation resulting from methacrylate and methacrylamide groups, was calculated to be 13.6% on molar bases, corresponding to 2.9% by weight, which can be attributed for ca. 0.82%_w/w_ to methacrylamide groups (deriving only from the 95% of lysine) and for ca. 2.05%_w/w_ to methacrylate groups (deriving from the 10.5% of with hydroxyproline, threonine, serine, and tyrosine OH residues).

### 3.2 Morphological and Ultrastructural Characterization of Biomaterials

After defining the methacrylation parameters, the optimized hydrogels (CollMA and PHNQs/CollMA) were produced and compared with non methacrylated collagen scaffolds (Coll). All lyophilized biomaterials formed 3D structures (Figure 4), with PHNQ-loaded samples displaying the characteristic red pigmentation. When dry, Coll scaffolds were softer and sponge-like, whereas CollMA and PHNQs/CollMA were more compact. These structural differences influenced hydration behavior: methacrylated hydrogels retained their shape and stability, while Coll scaffolds lost their structural integrity. CollMA and PHNQs/CollMA hydrogel formation was successful as these biomaterials absorbed water after rehydration, while Coll losed its structure with an evident structural collapse.

**Figure 4:**
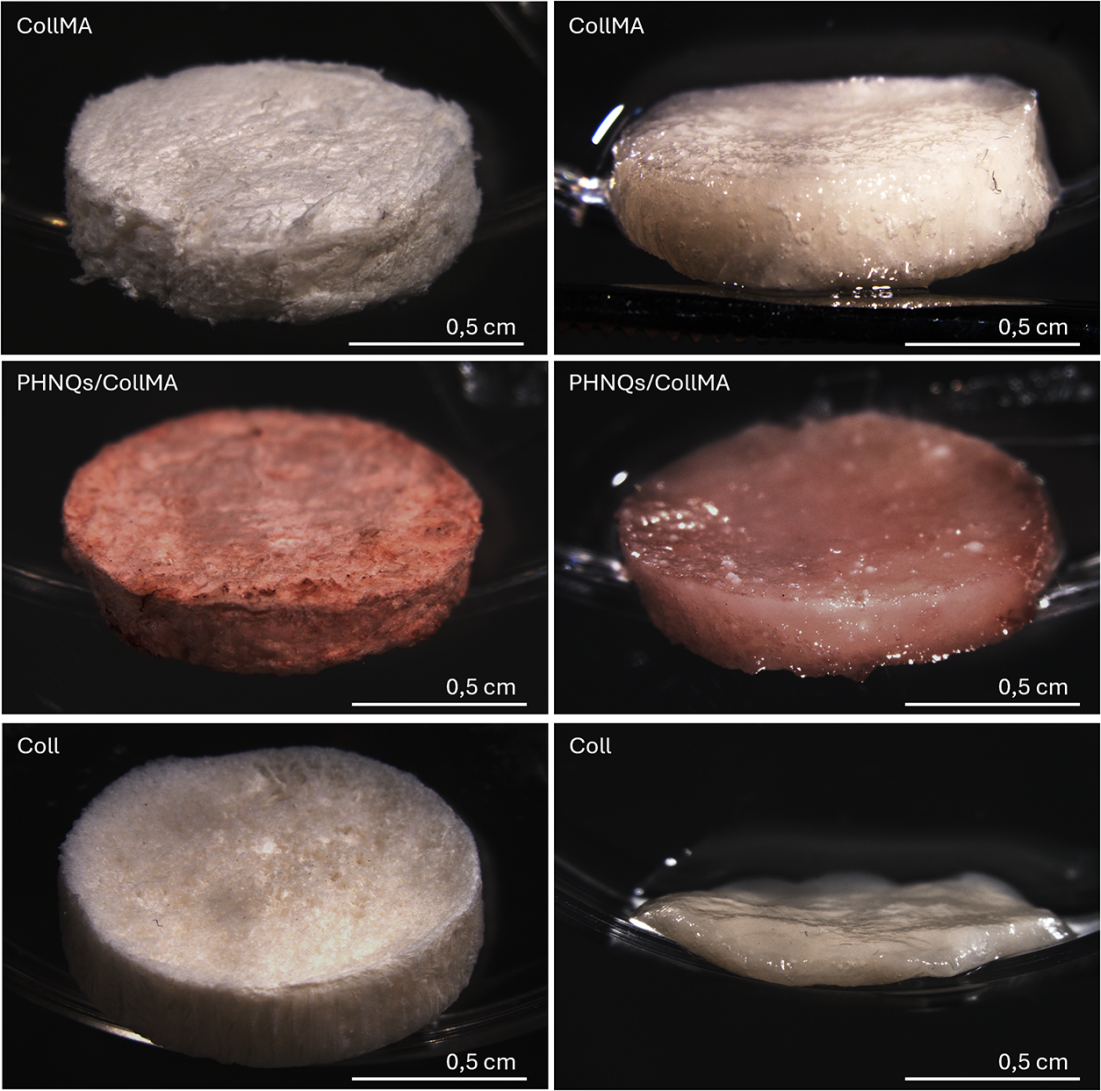
Stereo images of dry (left) and wet (right) methacrylated collagen hydrogels (CollMA and PHNQs/CollMA) and control collagen scaffold (Coll).

Scanning Electron Microscopy (SEM) analyses showed that the organization of the collagen fibrils in the lyophilized samples appeared homogeneous in all biomaterials. However, while the CollMA hydrogels and the Coll scaffolds showed a distinct fibrillar network, the PHNQs/CollMA hydrogels showed fibrils so closely adhered together that they appeared to form a single continuous laminar structure (Figure 5). Although laminar-like structures were also visible in Coll scaffolds (Figure 5E), they were clearly formed by tightly packed fibrils when observed at high magnification (Figure 5F), whereas in PHNQs/CollMA the laminar structures consisted of fibrillar networks embedded in a thin coating, probably formed due to the presence of a layers of PHNQ molecules covering part of the network. Preliminary observations indicated that the methacrylation step did not alter the structural organization of the collagen fibrils, including the D-patterning (Figure S1).

**Figure 5:**
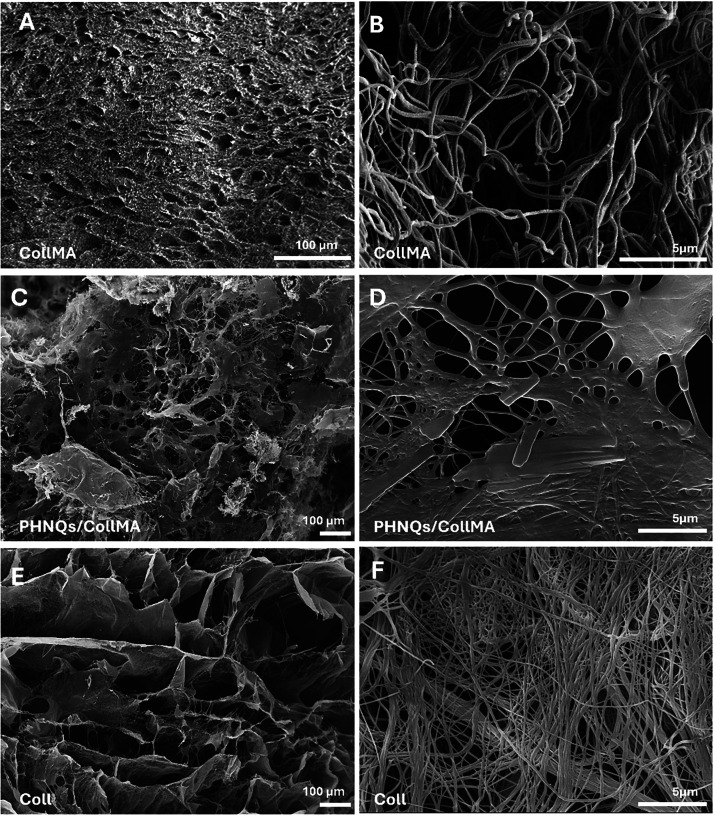
SEM images showing the ultrastructure of lyophilized methacrylated hydrogels: (A, B) CollMA, (C, D) PHNQs/CollMA, (E, F) lyophilized standard collagen scaffolds (Coll). Left: general overview; right: fibril details.

### 3.3 Macroporosity

Upon hydration all the tested biomaterials showed high macroporosity, although differences could be found among them (Figure 6). Particularly the methacrylated hydrogels showed significantly lower values than the control scaffolds (PHNQs/CollMA: 70.2% ± 0.7 (mean ± st. dev); CollMA 80.5% ± 1.0; Coll 85.5% ± 3.8; ANOVA with Tukey test: *p* < 0.01 CollMA *vs* Coll; *p* < 0.001 PHNQs/CollMA *vs* Coll), with the PHNQ loaded hydrogels displaying a significantly lower value than the methacrylated counterpart (ANOVA with Tukey test: *p* < 0.001 CollMA *vs* PHNQs/CollMA).

**Figure 6:**
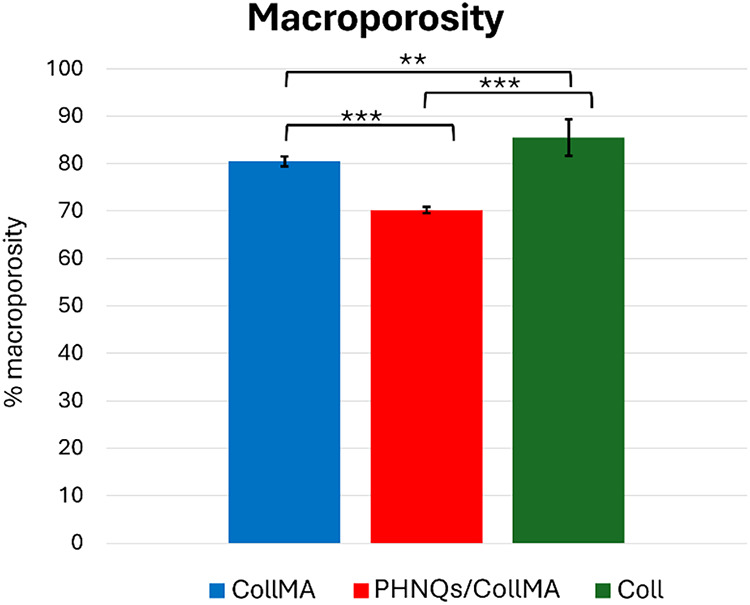
Methacrylated hydrogels (CollMA and PHNQs/CollMA) and standard collagen scaffolds (Coll) mean macroporosity values (%) (means ± st.dev). ** *p* < 0.01 and ***p < 0.001, One-Way ANOVA + Tukey test.

### 3.4 Swelling and Water Uptake

The swelling behavior was evaluated to assess any dimensional change in the structure of biomaterial in aqueous environment. Overall, hydrogels exhibited greater stability than Coll, but differences emerged depending on the presence of PHNQs. Representative images of samples before and after hydration are shown in Figure 4. Upon hydration, CollMA showed an area reduction after 3 h (−16.40 ± 0.03%, Figure 7A), whereas PHNQs/CollMA and Coll increased in area (+19.79 ± 0.08% and +39.0 ± 0.2%, respectively). In contrast, thickness variation displayed the opposite trend (Figure 7B): CollMA increased in thickness (+29.2 ± 0.2%), while PHNQs/CollMA and Coll showed a collapse, with decreases of −13.67 ± 0.04% and −73.10 ± 0.09%, respectively.

**Figure 7:**
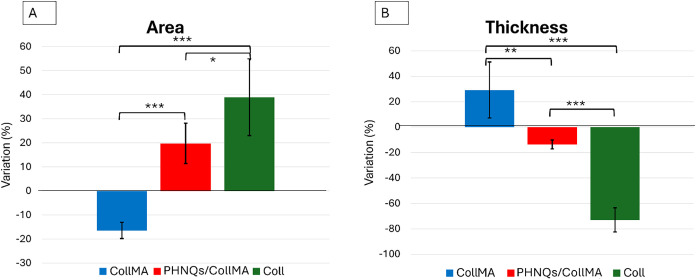
Bar plots showing in graph (A) the mean percentage of area variation and in graph (B) the percentage of thickness variation after 3 h hydration for hydrogels (CollMA and PHNQs/CollMA) and controls scaffolds (Coll); errors bars show the standard deviation. * *p* < 0.05, ** *p* < 0.01 and *** *p* < 0.001 (One-Way ANOVA + Tukey test).

Thickness and area variations between CollMA and Coll were statistically significant (*p* < 0.001, one-way ANOVA with Tukey test), confirming the superior stability of methacrylated hydrogels. PHNQs/CollMA also differed significantly from Coll (area: *p* < 0.05; thickness: *p* < 0.001, Tukey test), and the two hydrogels were significantly different from each other (area: *p* < 0.001; thickness: *p* < 0.01, Tukey test).

Water uptake, related to absorbed water, was assessed to mimic the wound environment. After 3 h (Figure 8) it reached about 1000% for both methacrylated hydrogels but remained significantly lower than for Coll (*p* < 0.05, Kruskal–Wallis + Dunn’s test). Values were 1064% ± 58 (CollMA), 1288% ± 197 (PHNQs/CollMA), and 11340% ± 755 (Coll).

**Figure 8:**
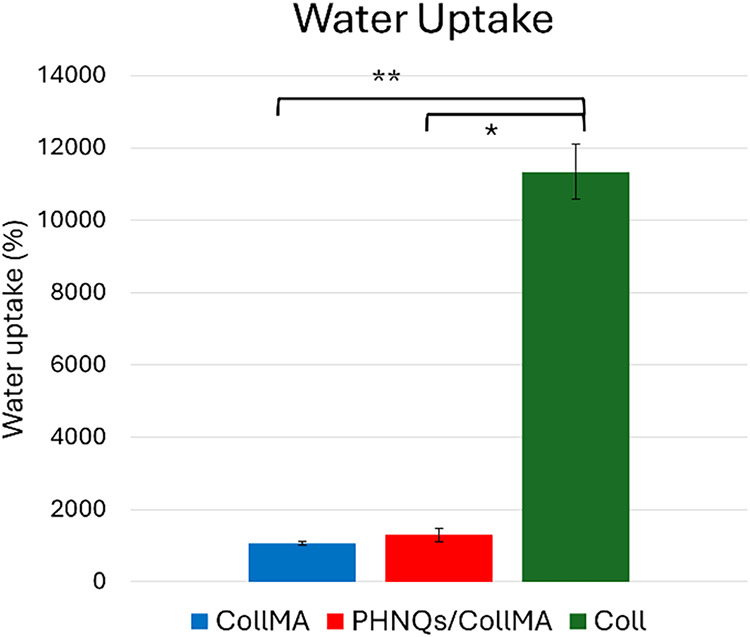
Bar plot showing the water uptake (expressed as means increase in weight (%) ± st.dv) for methacrylated hydrogels (CollMA and PHNQs/CollMA) and standard collagen scaffolds (Coll) after 3 h of hydration; errors bars show the standard deviation, **: *p* < 0.01; *: *p* < 0.05 (Kruskal–Wallis + Dunn’s test).

### 3.5 Degradation Kinetics

In PBS (Figure 9A), Coll degraded markedly faster: after 10 days, its remaining mass was 8 ± 8%, significantly lower than both CollMA (28 ± 3%, *p* < 0.05, MANOVA) and PHNQs/CollMA (47 ± 3%, *p* < 0.001). PHNQs/CollMA also showed significantly slower degradation than CollMA (*p* < 0.05).

**Figure 9:**
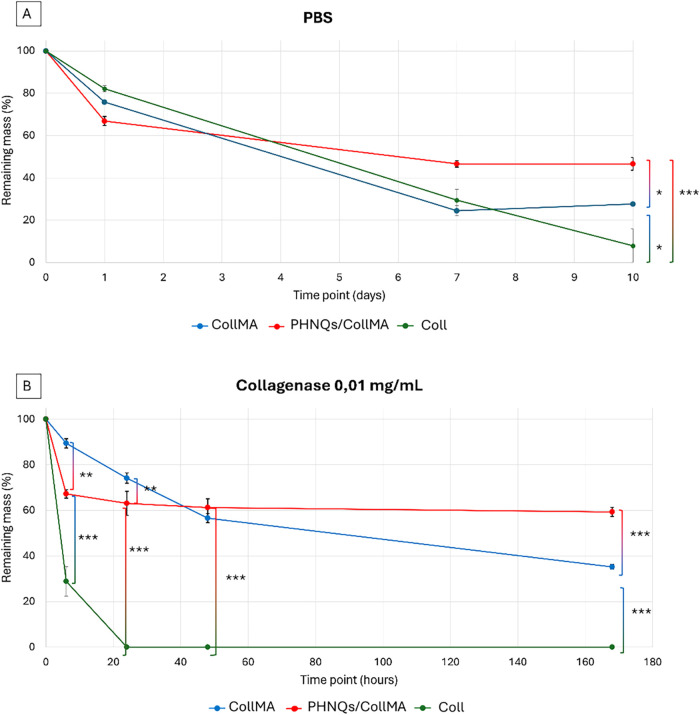
(A) Degradation curves in PBS for hydrogels (CollMA and PHNQs/CollMA) and standard collagen scaffolds (coll). Dots represent means ± standard error (bars), * *p* < 0.05, **p < 0.01, ***p < 0.001 (Manova test). (B) Degradation curves in collagenase solution for the hydrogels (CollMA and PHNQs/CollMA) and standard collagen scaffolds (Coll). Dots represents means ± standard error (bars), * *p* < 0.05, ** *p* < 0.01, *** *p* < 0.001 (Manova test).

Under enzymatic degradation (Figure 9B), hydrogels similarly showed greater stability than Coll. After 24 h in collagenase, Coll rapidly lost mass, whereas CollMA and PHNQs/CollMA retained 74 ± 2% and 63 ± 5, respectively. Differences between the two hydrogels became more evident over time: after 7 days, CollMA retained 35 ± 1% of its initial mass, while PHNQs/CollMA retained 59 ± 2% (*p* < 0.05, MANOVA).

### 3.6 Hydrogels PHNQs/CollMA Antioxidant Activity

At *t* = 0, PHNQs/CollMA hydrogels showed an EC_50_ of 0.0074 mg/mL, whereas no EC_50_ could be calculated for CollMA within the tested range (Figures 10 and S5), confirming that PHNQ loading provides antioxidant activity. The PHNQs/CollMA hydrogels (*t* = 0) displayed an EC_50_ comparable to that of a PBS solution containing the same PHNQ concentration (0.0078 mg/mL), indicating preservation of PHNQ functionality after incorporation. After 1 day in PBS, antioxidant activity remained similar (EC_50_: 0.006 mg/mL), but by day 10 no EC_50_ was reached, indicating a loss of antioxidant activity from the biomaterial.

**Figure 10:**
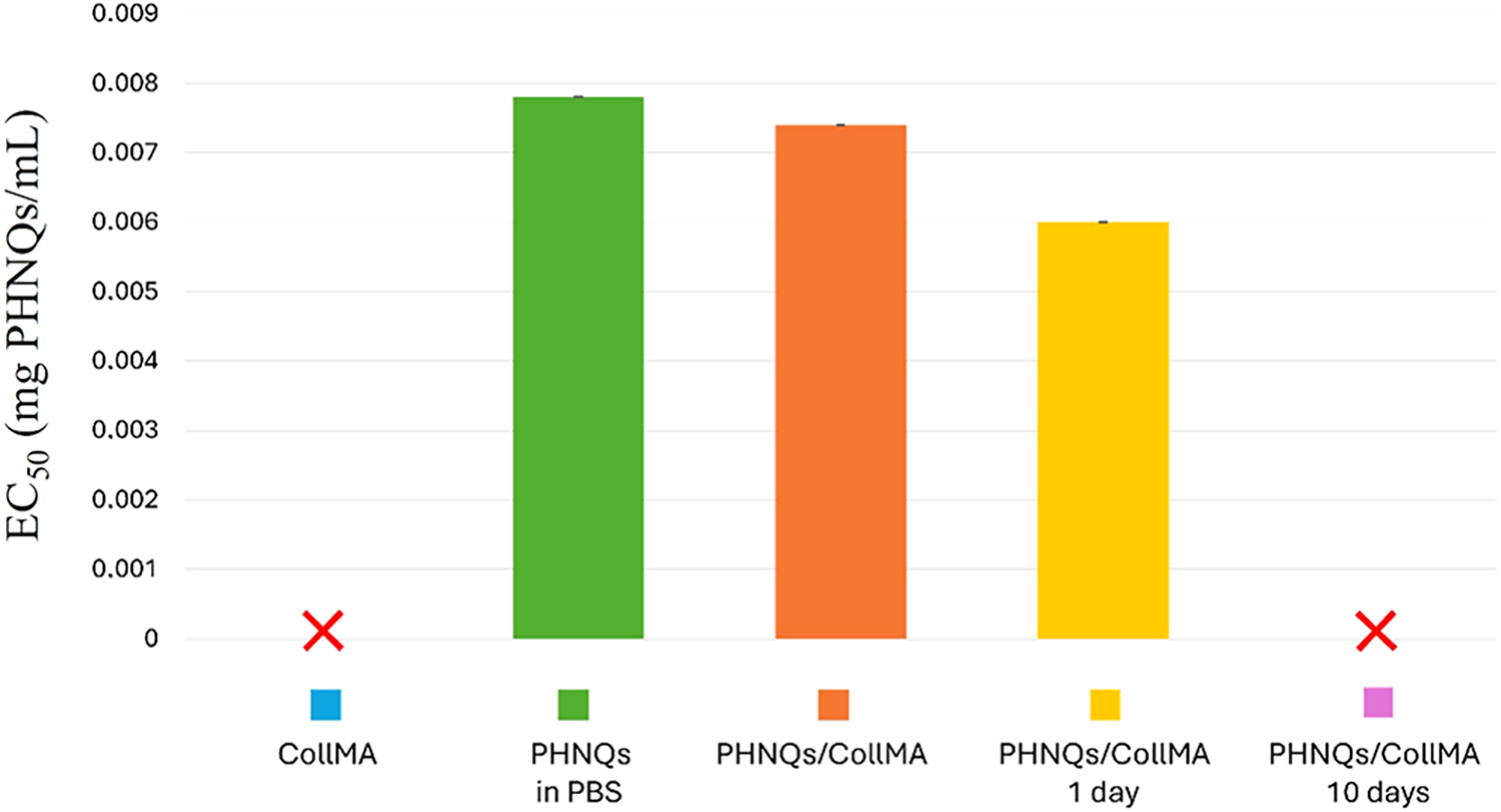
EC_50_ values of CollMA, PHNQs in PBS, and PHNQs/CollMA at time 0, 1 day and 10 days. No EC_50_ value was observed within the tested concentration range in CollMA and PHNQs/CollMA 10 day, showing low antioxidant activity.

### 3.7 Mechanical Tests

Table 3 displays the mechanical parameters obtained from the two-cycle compression test performed on the scaffolds after swelling. While the scaffolds made of pristine collagen (Coll) did not withstand contact with distilled water and collapsed, CollMA and PHNQs/CollMA retained their 3D network, allowing the compression tests to be performed. PHNQs/CollMA specimens exhibited higher compressive resistance and better elastic recovery than CollMA samples. In particular, the *F*_max_ value was approximately 1.5 times higher for PHNQs/CollMA scaffolds. However, this value was significantly lower than that obtained from the same foams conditioned at 80% relative humidity (*i.e.*, not swollen by direct contact with water), which exhibited an *F*_max_ of 0.114 N.^38^ Regarding elastic recovery, PHNQs/CollMA hydrogels also performed better, showing a higher recovery than CollMA foams (96.06% *vs* 80.64%, respectively).

**Table 3:** Resistance to Compressive Stress (*R*_max_, N) and Elastic Recovery (*E*_rec_, J/m^2^) for the Samples Coll-MA and Coll-MA PHNQ after Two Consecutive Compressive Cycles

sample	*F*_max_ (N)	*E*_rec_ (J/m^2^)^a^
CollMA	0.054 ± 0.002	–0.0085 ± 0.0007
PHNQs/CollMA	0.078 ± 0.003	–0.0033 ± 0.0004

a The sign minus refers to a loss, *i.e.*, the amount of compressive stress not recovered by the sample.

### 3.8 Cytocompatibility Test and Cell Morphology

Cells showed to be metabolically active, thus viable, once seeded with the biomaterial up to 7 days in culture were seeded with both hydrogels formulations (Figure 11A,B). The fluorescence intensity gradually increased from day 1 to day 7 and a statistically significant difference was observed at each time point. Cell metabolic activity in CollMA (Figure 11B), although increasing over time, suggested that the material could benefit from further optimization of its formulation. Indeed, when NDHF were grown into a low-methacrylation variant of the hydrogel (CollMA2), an increased metabolic activity was observed at all time points, with a higher fluorescent signal compared to cells cultured with CollMA (Figure 11A,B). CollMA2, due to the lower methacrylic anhydride content, did not form a three-dimensional hydrogel, but a stable thin film that remained intact in aqueous conditions.

**Figure 11:**
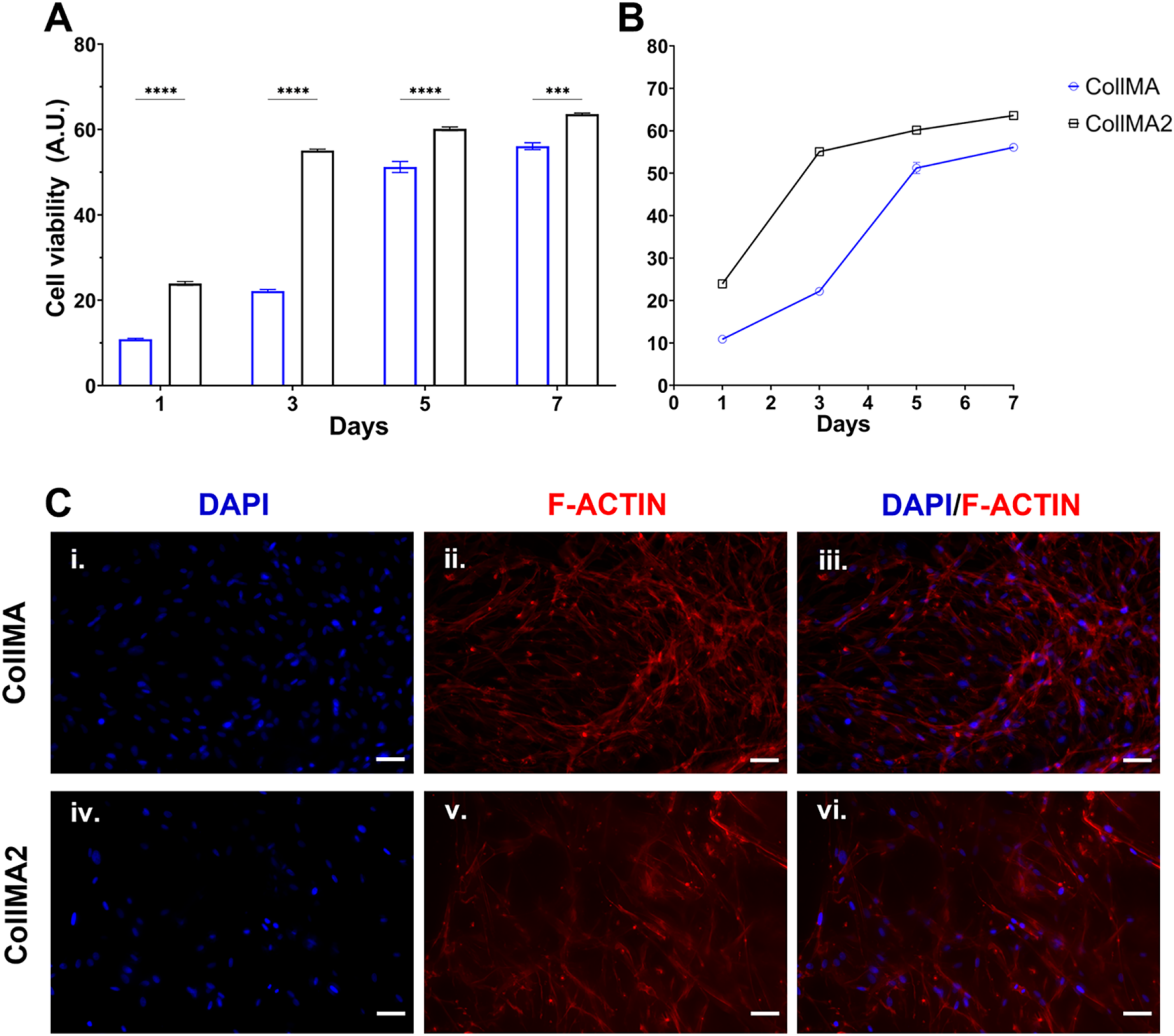
*In vitro* cytocompatibility of CollMA and CollMA2 biomaterials. (A) Cell viability (RFU) on both hydrogels showed increasing metabolic activity from day 1 to day 7. (B) Metabolic activity of cells cultured over time. (C) Representative microphotographs of cells stained for f-actin filaments (TRITC-conjugated phalloidin) and nuclei (Hoechst 33342) after seeding onto CollMA (i–iii) or CollMA2 (iv–vi); scalebar = 100 μm. Data are expressed as mean ± SD; *** *p* < 0.001, **** *p* < 0.0001.

The staining of cytoskeletal filaments and nuclei highlighted a fibroblast-like shape, with cytoplasmatic protrusion. This morphology was observed in both hydrogel formulations; however, on the CollMA hydrogels it was possible to appreciate the presence of cells on different planes (Figure 11C).

## 4 Discussion

Sea urchin collagen recovered from food waste represents a sustainable feedstock alternative to mammalian collagen, allowing to decouple collagen biomaterials from livestock-derived supply chains. This alternative sourcing potentially reduces environmental impact and supports circular-economy strategies while enabling new biomedical applications.^15,16,18^

To broaden usability beyond standard collagen scaffolds, we established for the first time a methacrylation protocol for native, fibrillar collagen in suspension, enabling photo-cross-linking and hydrogel formation while fully preserving the supramolecular organization of collagen. Indeed, the latter is characterized by a complex hierarchical organization: three α-chains (peptide level) form a triple-helical tropocollagen molecule (molecular level), which self-assembles into staggered fibrils (supramolecular level); the fibrils in turn aggregate into fibers (microscale level) and higher-order structures that are spatially organized within tissues.^66^ Although numerous collagen methacrylation protocols exist,^46–48,67,68^ they typically involve gelatin (denatured collagen), *i.e.*, disordered α-chains^69,70^ or acid-solubilized collagen (dispersed tropocollagen molecules).^46,71^ To our knowledge, methacrylation of native, fully preserved collagen fibrils has not been reported yet. Since the reaction occurs on supramolecularly organized fibrils rather than molecularly dispersed collagen (solubilized tropocollagen molecules or disperse polypeptide chains), mass transfer is limited, and reaction kinetics are slower. After optimization trials involving changes in reaction parameters (*i.e.*, collagen concentration, ionic strength, amount of methacrylic anhydride, reaction time), the best set was identified. These parameters were selected because they critically influenced the process of methacrylation in the final scaffold production. Collagen suspension concentration and Na_2_HPO_4_ molarity affect the accessibility of reactive groups and the pH, while the amount of methacrylic anhydride and the reaction time determine the degree of functionalization. Importantly, it was previously confirmed and reported in our recent work^72^ that methacrylation preserved the characteristic D-banding pattern of collagen fibrils, a ultrastructural feature arising from the native tropocollagen molecules, regarded as an indicator of intact molecular organization and critical for maintaining the mechanical integrity and biological functionality of collagen-based matrices.

ATR-FTIR analysis indicated modifications in the collagen structure consistent with the presence of methaclylamide and methacrylate groups however, the evidence was insufficient to provide further insight into the extent of methacrylation. Coherently with the literature, Han and co-workers reported negligible ATR-FTIR variations when methacrylic anhydride reacted with less than 5% of collagen amino acid residues.^63^

Confirming the indications provided by ATR-FTIR analysis, ^13^C CPMAS NMR spectra further evidenced structural modifications of collagen associated with the introduction of methacrylamide/methacrylate groups. In particular, after reaction with methacrylic anhydride, new signals appeared in the aliphatic region, attributable to acrylic methyl groups grafted onto amino acid residues. Following UV irradiation, the broadening and overlapping of amino acid resonances indicated reduced molecular mobility and increased system rigidity, consistent with the formation of a cross-linked methacrylated network. Moreover, the shift observed for part of the carbonyl signals further supported the occurrence of chemical and structural changes induced by the methacrylation process.

Following the qualitative confirmation of collagen methacrylation obtained by ATR-FTIR and solid state NMR analyses, the degree of functionalization was quantitatively evaluated by means of ninhydrin assay and ^1^H NMR spectroscopy. These complementary approaches provided information on the availability of reactive groups and, therefore, into the potential cross-linking capability of the system. Since both techniques required solubilized samples, Coll and CollMA were preliminarily hydrolyzed to obtain analyzable solutions. It should be noted that the hydrolysis treatment likely exposed additional amine groups that were not accessible for methacrylation in the native collagen suspension. Therefore, the quantitative values obtained from ninhydrin assay and ^1^H NMR analysis should be interpreted with caution, as they may not fully reflect the actual extent of functionalization occurring in the nonhydrolyzed collagen system.

The ninhydrin assay revealed a relatively low degree of functionalization (7% _w/w_), consistent with the intrinsically low lysine content of sea urchin-derived collagen (0.86%).^38^ Lysine residues represent in fact the only amino acids bearing amine groups available for methacrylamide formation (as demonstrated in the reactivity of single amino acids).

^1^H NMR analysis demonstrated that methacrylation was not limited to amine-containing residues. In addition to the signals assigned to methacrylamide groups derived from lysine, distinct olefinic resonances associated with methacrylate groups were identified, indicating the involvement of hydroxyl-bearing amino acids such as hydroxyproline, serine, threonine, and tyrosine. Quantitative evaluation showed that more than 95% of lysine residues were functionalized, whereas only about 10.5% of the available hydroxyl groups reacted with methacrylic anhydride. This difference reflects the markedly higher nucleophilicity of amine groups compared to hydroxyl groups, despite the much larger abundance of the latter within the collagen sequence.

However, the observed functionalization behavior cannot be explained solely by the intrinsic chemical reactivity of the amino acid residues. In the present system, methacrylation was performed on collagen fibrils dispersed in suspension rather than on solutions, as commonly occurs for methacrylated gelatin. Consequently, mass transfer limitations and steric hindrance likely reduced the accessibility of methacrylic anhydride to reactive sites located within the fibrillar structure, further limiting the overall functionalization efficiency. In addition, the subsequent photo-cross-linking process was probably affected by the restricted mobility of the fibrillar network. The formation of covalent bridges between relatively immobile collagen chains may reduce the probability of effective intermolecular cross-linking, thereby slowing down gelation kinetics and requiring longer UV exposure times. Therefore, both the limited degree of methacrylation and the structural constraints imposed by the fibrillar organization contributed to the observed cross-linking behavior. Although moderate, the achieved level of functionalization was nevertheless sufficient to enable the formation of a stable photo-cross-linked network, as supported by the spectroscopic evidence and the increased rigidity observed after a moderately long UV curing period.

The resulting hydrogels (CollMA and PHNQs/CollMA) were evaluated and compared to standard collagen scaffolds (Coll) to assess their physicochemical properties and stability, which are key parameters to assess preliminary a potential use in biomedical applications.

SEM revealed a comparable fibril network across all biomaterials (Figure 5) and a clear banding pattern of functionalized fibrils (Figure S1), indicating that methacrylation preserves the collagen native structure essential for biological function.^31,66,73^ This is consistent with a recent work from the same group and with reports showing that moderate methacrylation preserves Type-I bovine collagen’s fibrillar network.^72,74^ Both hydrogels exhibited macroporosity around or above 70% (Figure 6), a range considered optimal for cell infiltration, mass transport, and release of bioactive factors.^75,76^

Water uptake is an important parameter in the characterization of biodegradable polymers, affecting degradation, swelling behavior and inducing changes in mechanical properties and in biological response.^77^ Hydration studies showed that CollMA and PHNQs/CollMA were structurally more stable than Coll after 3 h (Figure 7), with Coll undergoing collapse. Similar stabilizing effect have been observed by Suvarnapathaki and colleagues in methacrylated gelatin (GelMA), where higher methacrylation restricts polymer chain mobility, resulting in a more stable and less deformable hydrogel network.^78^ Interestingly, while CollMA exhibited reduced swelling, shrinking in area and increasing in thickness, PHNQs/CollMA behaved oppositely, expanding laterally while thinning. This suggests a different network organization, likely due to PHNQs-mediated modulation of intermolecular interactions. These differences indicate that swelling degree and macroscopic deformation are strongly governed by cross-linking density and network architecture.^79^ In particular, macroporosity and network connectivity play a crucial role in regulating water uptake, as larger or more interconnected pores facilitate fluid absorption and transport, directly impacting hydrogel mechanics and stability. Comparable modulation of cross-linking by phenolic compounds has been observed by Fan and co-workers, as tannic acid can form hydrogen-bond and noncovalent interactions with polymer chains, strengthening cohesion and altering mechanical behavior in hydrogels.^80^

Water uptake was significantly lower in hydrogels than in Coll (Figure 8), reflecting the stabilizing effect of the cross-linked methacrylate network, which promotes controlled, anisotropic swelling consistent with other collagen hydrogels reported in the literature.^81,82^ This parameter is particularly relevant for biomedical applications, as controlled water uptake is essential to preserve structural integrity while ensuring adequate diffusion of nutrients, oxygen, and metabolites within the scaffold. This difference is particularly evident when considering that the swelling value measured for Coll (>11000%) is unusually high and not commonly reported for collagen-based systems, thus requiring careful clarification. Despite the precaution of removing water excess with adorbet paper, the material retains a substantial amount of water due to its highly porous and loosely organized structure, making complete removal of unbound water inherently difficult and potentially contributing to an overestimation of the swelling ratio.

The reduced swelling observed in CollMA hydrogels, on the other side, indicates a more organized and mechanically stable network, capable of maintaining its architecture under physiological conditions. In contrast, the non-cross-linked, non methacrylated Coll scaffolds lack sufficient network cohesion and structural reinforcement, leading to uncontrolled (isotropic) swelling followed by collapse. This behavior is typically associated with poorly defined macroporosity and weak intermolecular interactions, which allow rapid and uncontrolled water penetration without mechanical resistance.

These results highlight how methacrylation and cross-linking govern not only the extent of swelling but also pore structure, network connectivity, and mechanical performance,^83–85^ key features for maintaining the shape and mechanical integrity of biomaterials *in vivo* where hydration levels fluctuate.^86^

Degradation assays further supported the enhanced stability of methacrylated hydrogels. In PBS, hydrogels degraded more slowly than Coll, although all biomaterials showed substantial mass loss after 10 days due to continuous immersion (Figure 9A), which accelerates degradation compared to the more limited fluid exposure typically encountered in physiological wound environments.

This improved stability can be attributed to the increased cross-linking density introduced by methacrylation, which results in a tighter polymer network that restricts water uptake and limits matrix swelling. Importantly, such network architecture can also reduce enzyme accessibility to cleavage sites within the collagen backbone. Steric hindrance, reduced pore size, and decreased diffusivity within the hydrogel matrix likely impair enzyme penetration and mobility, thereby slowing enzymatic degradation kinetics. In contrast, the native collagen (Coll), characterized by a more open and less cross-linked structure, allows easier enzyme infiltration and interaction with susceptible sites, resulting in faster degradation.

Therefore, the observed degradation behavior reflects not only intrinsic material stability but also physicochemical constraints on enzyme–substrate interactions, which are critical in determining degradation profiles in biologically relevant conditions.

In collagenase, Coll was fully degraded within 24 h, whereas CollMA and PHNQs/CollMA retained mass at both 24 h and 7 days (Figure 9B). This increased enzymatic resistance is consistent with the presence of cross-links that strengthen the network.^87,88^ In general, the resistance of hydrogels to enzymatic degradation makes them suitable for high protease environments such as chronic wounds or inflammatory sites,^89^ to support tissue regeneration over an extended period of time^29,30^ or as a delivery system for sustained release of bioactive molecules.^90–92^

PHNQs/CollMA showed the lowest degradation rate under both conditions, consistent with previous observations in PHNQ-containing collagen scaffolds,^38^ suggesting that PHNQs contribute to hydrogel stabilization. The role of polyhydroxynaphthoquinones (PHNQs) in modulating collagen-based materials has already been extensively investigated in our previous work by Martinelli et al.^38^ In that study, the incorporation of PHNQs into collagen matrices was shown to significantly enhance hydrogel stability and structural integrity, leading to slower degradation kinetics and improved mechanical performance compared to pristine collagen. These effects were attributed to strong interactions between collagen and PHNQs, further supported by computational analyses suggesting the possible formation of covalent bonds between PHNQ molecules and collagen chains (interaction between the tripeptide glycine-arginine-aspartic acid spinochrome A). In the present system, although collagen has undergone methacrylation, the majority of reactive sites potentially involved in PHNQ interactions remain available, as the functionalization selectively targets a limited fraction of lysine residues. Therefore, it is reasonable to expect that PHNQs retain similar reactivity toward CollMA, likely providing an additional stabilizing contribution to the fibrillar network and further reinforcing the overall hydrogel structure.

Beyond stability, PHNQs conferred antioxidant functionality to the hydrogels. The PHNQs/CollMA hydrogel displayed antioxidant activity comparable to Trolox an analogue of vitamin E commonly used in the literature as a reference antioxidant.^93^ The EC50 value was also comparable with that of PHNQs in solution at time 0, indicating no loss of PHNQs functionality during preparation (Figure 10B).^93^ Most antioxidant capacity persisted after 24 h in PBS, which is relevant for early wound-healing stages characterized by high oxidative stress.^94^

Finally, mechanical tests results further support the beneficial role both of methacrylation and PHNQs in reinforcing the collagen-based network and improving the structural stability of the scaffolds under hydrated conditions. The increase in *F*_max_ parameter suggests, and confirm, that intermolecular interactions between collagen and PHNQs contribute to a more cohesive and mechanically robust network, while preserving excellent resilience, recovering up to 96% of their original shape after compression.

When running *in vitro* studies, an additional biomaterial with a lower degree of methacrylation (CollMA2) was prepared to further investigate the effect of cross-linking density on cell response. Results indicated that lower methacrylation (CollMA2) enhanced metabolic activity, suggesting improved cytocompatibility by reducing acidic byproducts. Methacrylation is in fact known to influence cell response by modulating network density and surface chemistry, which in turn regulate cell adhesion, proliferation, and morphology.^95^ However, despite the improved metabolic activity, this strategy proved not to be a viable solution in the present system, as the resulting material exhibited limited structural performance. In particular, the reduced degree of methacrylation prevented full hydrogel formation, leading to the formation of thin films rather than stable three-dimensional matrices, which are required for the intended applications. This might also have influenced cell viability, as a three-dimensional environment of the hydrogel could have hindered cell viability due to a physical confinement so that cells were rather growing and migrating onto a 2D matrix than increasing in number during the first days in culture.^96^ Comparable trade-offs have been reported in GelMA, where excessive cross-linking (higher methacrylation) significantly impairs cell viability by increasing stiffness and reducing cell spreading.^97^ Nonetheless, it can be hypothesized that the first 3 days in culture might be an “adaptation” phase for cells to the 3D structure of the hydrogel, hence allowing a proper migration, adhesion, and spread. Following this phase, at day 5 cells showed an increased metabolic activity, thus an increased number due to cell proliferation. Moreover, the presence of cells was confirmed by visual observation of fluorescent images, confirming the fibroblast morphology; furthermore, cell morphology did not appear to be affected by the different hydrogel formulations. Together, these findings indicate that an optimal formulation may lie between the two extremes, retaining the improved cellular response of CollMA2 while restoring the hydrogel-forming ability characteristic of CollMA. Future work will therefore focus on fine-tuning the methacrylation level to achieve a balance between structural integrity and biological performance.

## 5 Conclusion

In conclusion, this work demonstrates for the first time the successful methacrylation and photo-cross-linking of native fibrillar collagen recovered from sea urchin food waste while preserving its hierarchical supramolecular organization. The developed strategy enabled the formation of stable collagen-based hydrogels with enhanced structural integrity, reduced swelling, improved resistance to hydrolytic and enzymatic degradation, and superior mechanical performance under hydrated conditions compared to non methacrylated collagen scaffolds. The incorporation of PHNQs further reinforced the hydrogel network, contributing not only to enhanced stability and elasticity through strong collagen–PHNQ interactions, but also imparting significant antioxidant activity, which is particularly attractive for applications in oxidative and inflammatory environments such as chronic wounds. Although the degree of methacrylation achieved was moderate due to the intrinsic complexity and limited accessibility of reactive sites within native collagen fibrils, it proved sufficient to obtain photo-cross-linked hydrogels while maintaining the characteristic fibrillar architecture essential for biological functionality. Preliminary *in vitro* studies highlighted the importance of balancing cross-linking density and cytocompatibility, suggesting that further optimization of the methacrylation level could improve cellular response while preserving hydrogel stability.

Benchmarking against mammalian-collagen commercial matrices (*e.g.*, Integra’s bovine collagen/chondroitin system) and against high-functionalization methacrylated gelatin systems (GelMA) highlights two advantages: (i) sustainability *via* waste-derived sourcing and reduced dependence on mammalian tissues, and (ii) functional performance *via* covalent stabilization of a native fibrillar network.

Commercially, methacrylated mammalian collagen products and kits are already available (*e.g.*, “methacrylated collagen hydrogel/bioink” offerings), underscoring demand for photo-cross-linkable collagen matrices. Our approach differs by targeting native fibrillar marine collagen in suspension, preserving the supramolecular organization and leveraging a sustainable sea food waste feedstock. In this sense, the key innovation is not only “collagen that crosslinks,” but “a waste-derived, fibrillar collagen that crosslinks,” enabling new design space for biomaterials where sustainability and functionality improve together rather than trading off.

Future studies should focus on tuning methacrylation to maximize cytocompatibility while preserving 3D gelation, and on validating *in vivo* biocompatibility, integration, and degradation in clinically relevant wound models.

## Supplementary Material



## Notes

Methacrylation reaction experimental trials; SEM image evidencing the d-patterning of CollMA; NMR results (free amino acids reactivity, degree of methacrylation); ABTS assay results (PDF)

## ⊥

Link Campus University, Department of Human Sciences, Via del Casale di San Pio V 44, 00165 Rome, Italy
