# Supplementary material for: Methacrylation of Fibrillar Sea Urchin Collagen: Production of Sustainable, Stable, and Functional Hydrogels
Source: ACS Omega. 2026 Jul 7;11(28):41896–913. doi: 10.1021/acsomega.6c01928 (PMC13393184; doi:10.1021/acsomega.6c01928)
Supplement: Supplementary file 1 [file ao6c01928_si_001.pdf]

SUPPORTING INFORMATION

Methacrylation of Fibrillar Sea Urchin Collagen: Production of Sustainable, Stable, and Functional Hydrogels

Margherita Roncoroni[1], *Giordana Martinelli*[1], *Tamara Chwojnik*[1], *Chiara Scapuzzi, Luca Melotti*[2], *Anna Carolo*[2], *Daniela Maggioni*[3], *Stefano Farris*[4], *Marco Patruno*[2], *Stefania Marzorati*[1,]*, *Michela Sugni*

[1] Department of Environmental Science and Policy, University of Milan, Via Celoria, 2, 20133, Milan, Italy.

[2] Department of Agronomy, Food, Natural Resources, Animals and Environment, University of Padua, Via dell'Università, 16, 35020 Legnaro (PD)

[3] Department of Chemistry, University of Milan, Via Golgi 19, 20133 Milan, Italy

[4] Department of Food, Environmental and Nutritional Sciences, University of Milan, Via Celoria, 2, 20133, Milan, Italy

Corresponding Author

stefania.marzorati@unimi.it

1. Methacrylation reaction optimization

Table S1. Reaction conditions tested in different methacrylation trials.

Tested Value					Result	Rationale
Collagen Concentration (mg/mL)	Na_2HPO_4 concentration in the solvent mixture (M)	Volume of Methacrylic anhydride MA (mL) per mL of collagen suspension	Reaction time (h)	Dialysis time (days)		
4	0	0.645	4	/	Rapid collagen aggregation during the reaction and no visible methacrylation at FTIR	Aggregation suggests excessive MA and/or unsuitable solvent → less MA were maintained and a buffer introduced. Reaction time and collagen concentration were increase
4	0	0.129	4	/	Collagen aggregation at the end of 4 hours and no visible methacrylation at FTIR	
4	0.05	0.129	6	/	Successful methacrylation but collagen aggregation after 2 hours	Aggregation suggests high buffer concentration is required
4	0.2	0.129	6	5	Successful methacrylation but collagen aggregation post dialysis	5 days dialysis proved insufficient → extended
5	0.2	0.129	4	10	Successful methacrylation but partial hydrogel formation	Optimized condition allowed efficient methacrylation and hydrogel formation
5	0.2	0.129	6	10	Successful methacrylation and proper hydrogel formation	

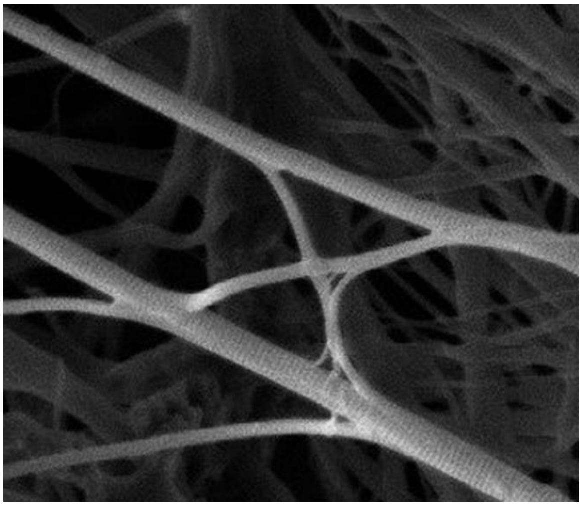

Figure S1. SEM image of the methacrylated collagen fibril.

2. NMR results

2.1 Free amino acids reactivity

By using high resolution NMR in solution we investigated on the reactivity of some amine containing groups among those amino acids present in our collagen: in order to better mimic the situation found in the collagen polypeptide chain, where the α-amino group is involved in peptide bonding and thus not reactive, each amino acid was reacted with two equivalents of MA. This ensured that the α-amino group was already functionalized, allowing the observation of the reactivity of side-chain amine groups only. For this purpose, lysine (**Figure S1**), arginine (**Figure S2**) and histidine were each reacted with methacrylic anhydride (MA) in a Na_2HPO_4 buffer solution. Specifically, 50 mg of each amino acid were dissolved in the buffer solution (pH=9) to obtain final concentrations of 34.20, 28.71 and 32.22 mM, respectively. MA at a molar ratio of 2:1 (MA:AA) was then added to the solution, which was stirred in the dark at room temperature for six hours. Solution spectra were acquired by adding 100 μL of D_2O using the *zgpr* Bruker sequence for the water suppression (Bruker DRX400 spectrometer equipped with a Bruker 5 mm BBI Z-gradient probe head with a maximum gradient strength of 53.5 G/cm (π/2 pulse: ^{1}H 8.5 μs)

operating at 400.13 MHz). The NH_2 group of lysine promptly reacts with the first MA equivalent as ascertained by the variation in chemical shift of CH(α) resonance from 3.62 to 4.15 ppm. Moreover, the second MA equivalent also easily reacts with the amine of the R group of the lysine, as stated by the CH_2(ε) resonance shift from 2.90 to 3.18 ppm. The appearance of two main amide signals at 7.89 (t) and 7.62 (d) ppm confirm the reaction on both the amine of lysine. On the contrary, despite the high basicity of the lateral group of arginine, ^{1}H NMR spectra showed that arginine does not react with MA by the guanide pendent, but only with the amine group, even at pH 12, due to the delocalization of the protonation of this functional group that, as expected, turns off its reactivity. Overall, these results suggest that, of the basic amino acids tested, lysine exhibits the greatest reactivity towards methacrylation due to the nucleophilicity of its α- and ε-amino groups. Arginine and histidine, on the other hand, show limited reactivity under the tested conditions, likely due to the protonation state or electronic delocalisation of their side chains. Considering the amino acid composition of our collagen (0.86 g lysine/100 g, 0.79 g histidine/100 g and 8.35 g arginine/100 g), arginine is the most abundant basic residue. While there was no clear evidence of reactivity of its guanidinium group in our model studies, its contribution to methacrylation in the full collagen matrix cannot be completely excluded.

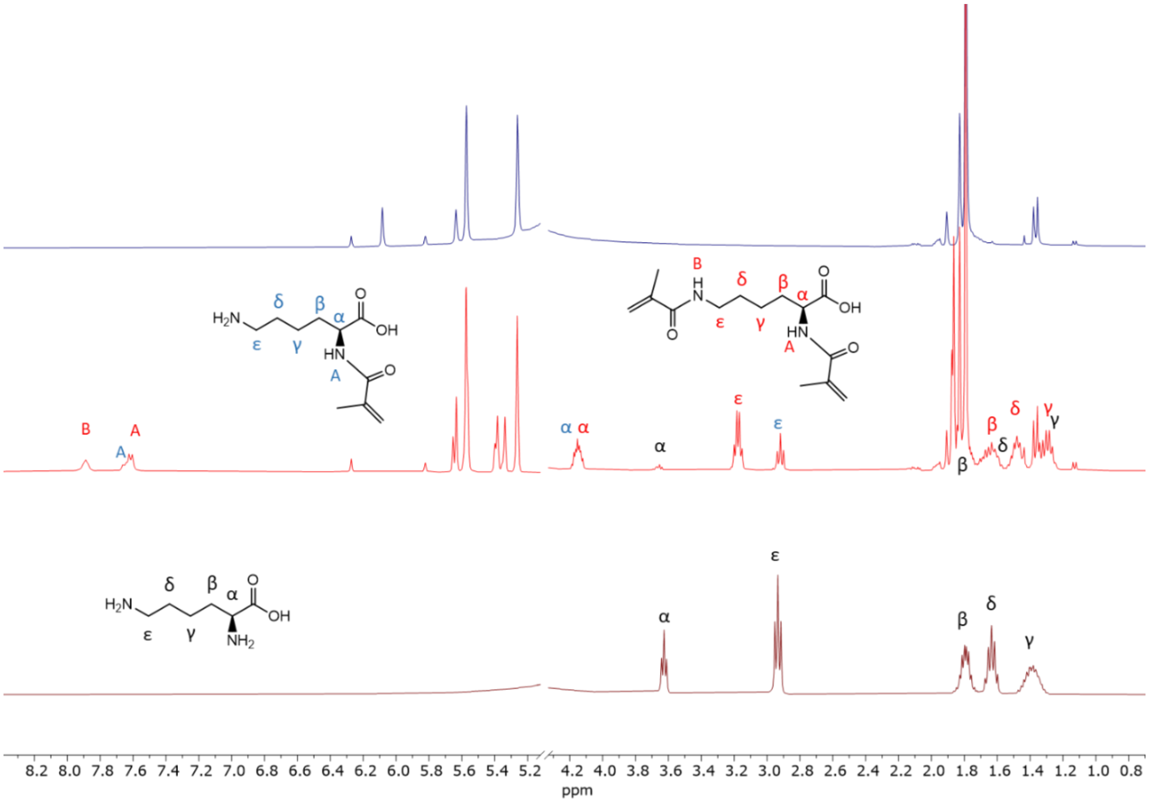

Figure S2. Stacked plot of ^{1}H NMR spectra of lysine (bottom), the mixture reaction of lysine and 2 equiv of MA, and MA (9.4 T, 300 K, H_2O/D_2O 9:1).

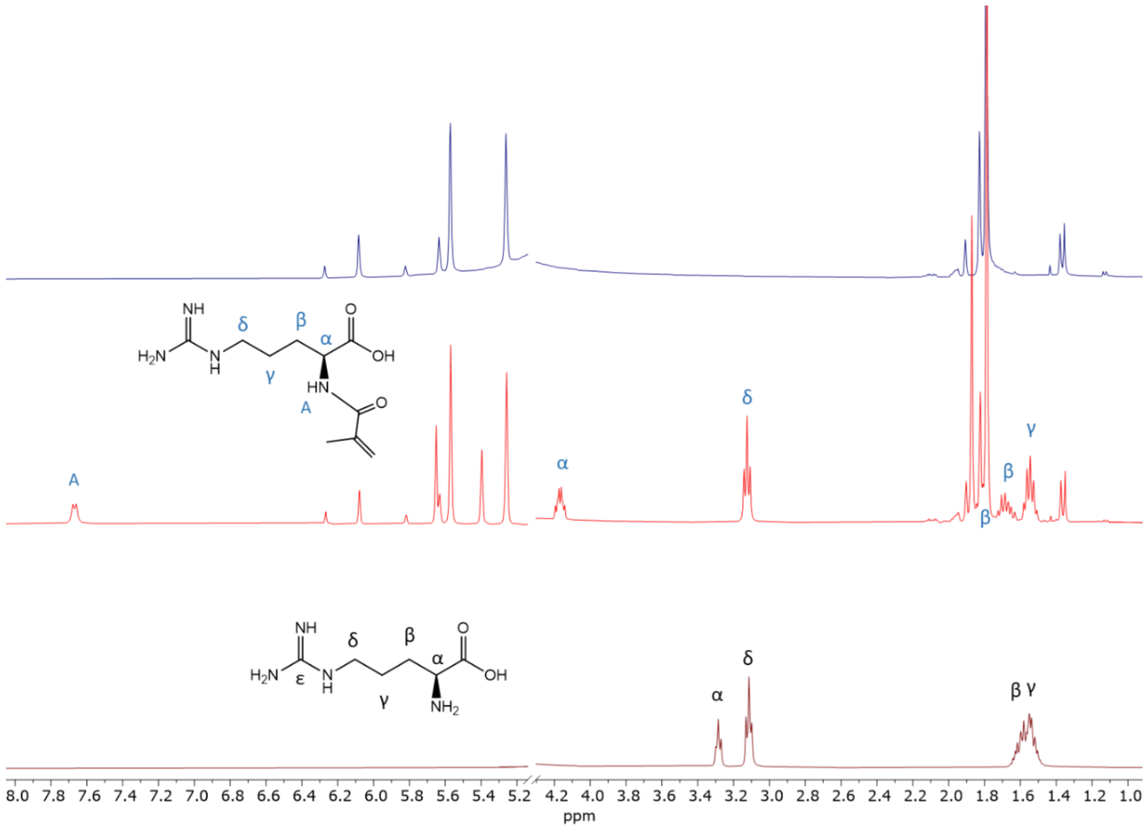

Figure S3. Stacked plot of ^{1}H NMR spectra of arginine (bottom), the mixture reaction of arginine and 2 equiv of MA, and MA (9.4 T, 300 K, H_2O/D_2O 9:1).

2.2 Degree of methacylation

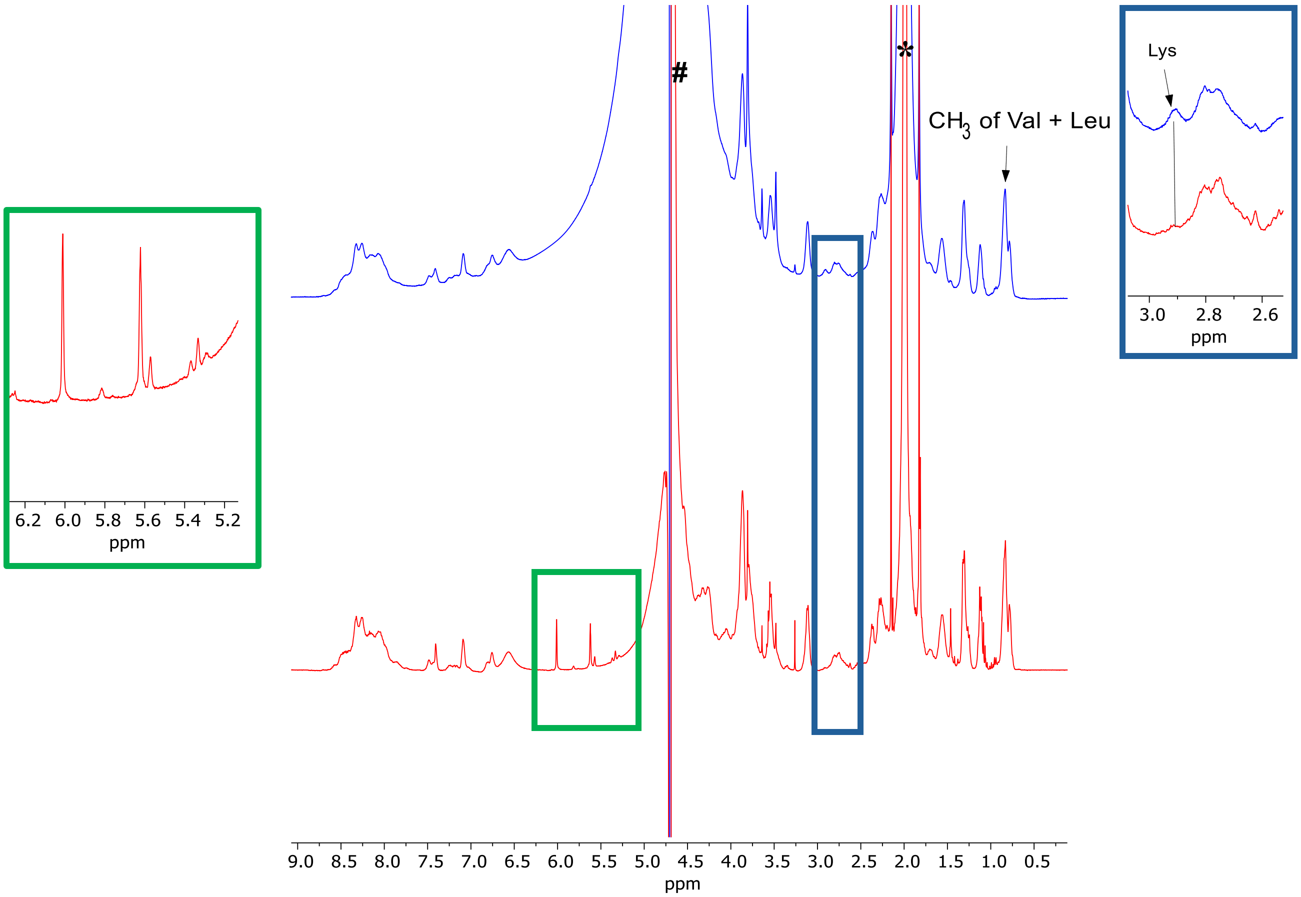

Figure S4. Stacked plot of ^{1}H NMR spectra of hydrolysed Coll (blue trace) and CollMA (red trace) (9.4 T, 300 K, H_2O/D_2O 9:1, zgpr Bruker sequence for the presaturation of the solvent #). The asterisk marks the methyl group of acetic acid. The green box highlights the alkene signals, while in the blue box it is evidenced the ε CH_2 signal of lysine that disappears after the reaction with methacylic anhydride to form an amide group.

Table S2. Composition of Coll in % by weight and the corresponding amount in mols of the only amino acids capable to react with methacrylic anhydride in the experimental conditions used in this work.

**Amino acid (aa)**	**mass (g) aa / 100 g Coll**	**mol aa / 100 g Coll**
Hydroxyproline	8.06 %	0.0615 mol
Threonine	3.74 %	0.0314 mol
Serine	6.15 %	0.0585 mol
Tyrosine	1.45 %	0.0080 mol
Lysine	0.86 %	0.588

2.3 Calculations for the determination of degree of methacrylation by high resolution ^{1}H NMR.

The integrated intensity (I) of the methyl signals of the amino acids leucine and valine (lying in the range 0.73–0.91 ppm) was used as an internal reference and arbitrarily set to 100, corresponding to the integration of 6 protons. Knowing the weight percentage of these two amino acids (2.96% and 2.50% w/w, respectively), the total corresponding amount can be calculated as 0.04390 mol.

Using these data, together with the integrated intensity of the olefin signal at 6.01 ppm (I = 6.37, 1H), a proportion can be established (100/6 : 0.04390 mol = 6.37 : x), from which the moles of methacrylate ester groups, formed by reaction of methacrylic anhydride with OH residues, are obtained, which are equal to 0.01678 mol.

Similarly, considering the integrated intensity of the signal at 5.57 ppm (I = 2.17, 1H), a proportion can be set with the moles corresponding only to lysine residues (100/6 : 0.04390 mol = 2.17 : x), allowing the calculation of methacrylamide moles, equal to 0.005715 mol.

By calculating the ratio between the moles of methacrylamide and the total moles of lysine, it results that more than 95 mol% of lysine residues have been methacrylated.

Regarding the calculation of the percentage of reacted OH groups, the ratio between the previously calculated moles derived from the integrated intensity (0.01678 mol) and the sum of the moles of

the four amino acids (hydroxyproline, threonine, serine, and tyrosine) containing OH functionalities (0.1594 mol) was considered. From this ratio, it follows that only 10.5 mol% of these functional groups have been methacrylated.

To compare the result obtained with the ninhydrin assay, the molar percentage was converted into weight percentage by considering a weighted average molecular weight (<MM> = 124.31 g/mol) for the four aforementioned amino acids, based on their relative abundance in collagen (see Table S2). This step is necessary because it is not possible to discriminate the individual contribution of these four amino acids from the NMR spectrum to the methacrylation. In this way, a corresponding methacrylation degree of 2.9 wt% is obtained.

3. ABTS results

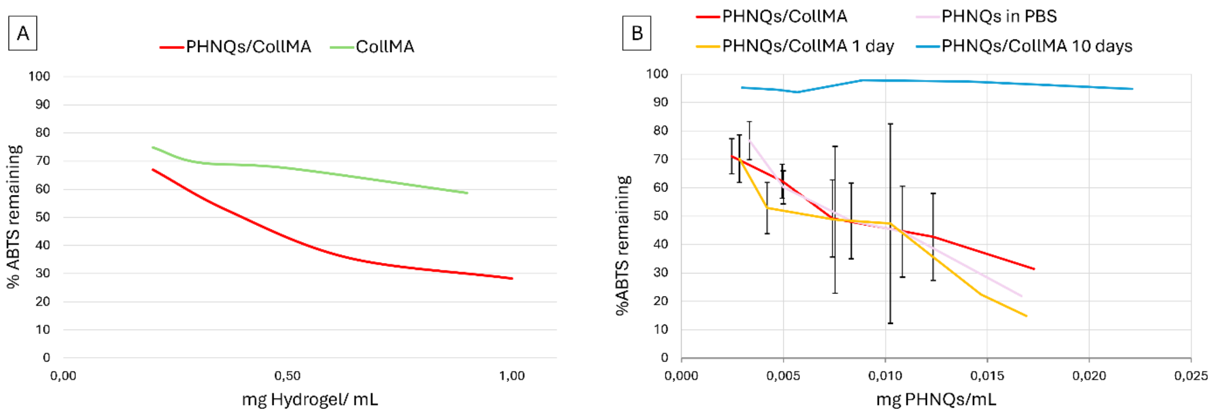

Figure S5: A) ABTS remaining (%) in PHNQs/CollMA (red) and CollMA (blue) hydrogels. The graph shows a higher ABTS degradation rate in PHNQs/CollMA compared to CollMA, suggesting differences in their antioxidant activity. B) ABTS remaining (%) in PHNQs/CollMA at different conditions - time 0 (red), after 1 day in PBS (yellow), after 10 days in PBS (blue) and the PBS solution containing the same amount of PHNQs as used in the hydrogel preparation (pink). The graph shows a similar ABTS degradation rate in all, except for hydrogels immersed in PBS for 10 days which showed a reduction in antioxidant activity.
